# Integrated Multiomics Analysis Reveals a Migrasome‐Related Signature for Prognosis and Immunotherapy Response in Lung Adenocarcinoma

**DOI:** 10.1155/humu/8778797

**Published:** 2026-01-08

**Authors:** Jiayu Zhou, Tianye Song, Nengzheng Wang, Ce Liang, Ming Jiang, Xu Zhang, Hong Gao, Qingqing Feng

**Affiliations:** ^1^ Department of Thoracic Surgery, Sir Run Run Shaw Hospital, School of Medicine, Zhejiang University, Hangzhou, China, srrsh.com; ^2^ Department of Thoracic Surgery, The Second Hospital of Hebei Medical University, Shijiazhuang, China, hebmu.edu.cn; ^3^ School of Life Sciences, Fudan University, Shanghai, China, fudan.edu.cn; ^4^ Center for Genetic Medicine and Department of Otolaryngology-Head and Neck Surgery, The Fourth Affiliated Hospital, Zhejiang University School of Medicine, Yiwu, China, zju.edu.cn; ^5^ Department of Oncology, The Second Hospital of Hebei Medical University, Shijiazhuang, China, hebmu.edu.cn; ^6^ Department of Pulmonary and Critical Care Medicine, Shanxi Provincial People’s Hospital, Taiyuan, China, spph-sx.com

**Keywords:** immunotherapy prediction, lung adenocarcinoma, machine learning, migrasomes, tumor microenvironment

## Abstract

**Background:**

Migrasomes, a newly identified subtype of extracellular vesicles generated during cell migration, play crucial roles in tumor microenvironment modulation. However, their systematic characterization in lung adenocarcinoma (LUAD) remains unexplored. This study is aimed at deciphering migrasome‐related molecular features and their clinical significance through multiomics integration.

**Methods:**

We integrated bulk transcriptomes (541 LUAD samples from TCGA/GEO) with single‐cell RNA‐seq (GSE156632). Migrasome‐related genes (MIGgenes) were identified through WGCNA and differential expression analysis. A machine learning framework incorporating 10 algorithms generated 101 combinatorial models, with the optimal prognostic signature (MIGsig) selected via 10‐fold cross‐validation. Biological mechanisms were investigated through ssGSEA, TME analysis, and in vitro validation.

**Results:**

Our analysis revealed significant migrasome activity enrichment in endothelial cells and fibroblasts, with 115 cross‐omics MIGgenes identified including 31 prognostic markers. The Lasso–Cox‐derived 3‐gene signature (GSTM5/DNASE1L3/PDGFB) demonstrated robust predictive performance (training set *C* index = 0.703; validation set GSE50081 AUC = 0.678). The low‐MIGsig group exhibited characteristic “hot tumor” features, including elevated immune infiltration and higher tumor mutational burden, and significantly improved immunotherapy response rates in the IMvigor210 cohort. Finally, MIGsig‐related genes were further validated by in vitro experiments and public database.

**Conclusions:**

This study establishes the first migrasome‐based prognostic model for LUAD, demonstrating both independent survival prediction capability and clinical utility for identifying immunotherapy beneficiaries. The MIGsig signature provides novel biological insights into migrasome‐mediated tumor–immune interactions and represents a promising tool for precision oncology applications in LUAD management.

## 1. Introduction

Extracellular vesicles (EVs) are ubiquitous components of human bodily fluids that significantly contribute to intercellular communication and material transfer [1]. Within the tumor microenvironment (TME), tumor‐derived EVs serve as pivotal mediators for local and systemic crosstalk between malignant cells and stromal elements [2]. These vesicles actively participate in driving both primary tumor development and metastatic progression through multiple pathophysiological mechanisms—such as immunomodulation, coagulation control, vascular permeability adjustment, and extracellular matrix reorganization—which collectively prime distant sites for metastatic invasion [3, 4]. The cargo composition of EVs includes diverse bioactive molecules comprising nucleic acids (e.g., DNA, mRNA, microRNA, and noncoding RNAs), proteins (including receptors, transcription factors, cytokines, enzymes, and extracellular matrix components), and lipid species, all capable of functionally reprogramming recipient cells [5]. As a well‐investigated EV subclass, exosomes have attracted considerable research attention for their specialized role in mediating material and signal exchange within the TME, profoundly impacting oncogenesis and metastatic dissemination [6].

Migrasomes are a newly discovered subcellular structure, first reported by Ma and colleagues in 2015. The formation of migrasomes depends on cell migration, serving as a novel mechanism for the extracellular release of cytoplasmic components [7]. During cellular movement, tubular structures called contractile actin cables form at the rear of the cell. As migration progresses, vesicle‐like structures emerge at the tail or intersections of these actin cables, gradually increasing in diameter to 1–3 *μ*m, constituting migrasomes. Studies indicate the presence of migrasomes in regions exhibiting cell migration across various organisms. After cell migration, migrasomes remain in situ until ruptured or engulfed by other cells, releasing the components of the donor cell [8]. Compared to exosomes, migrasomes differ in size, ultrastructure, generation mechanism, membrane composition, and content, representing a distinct type of EV‐like structure [9]. In the context of cancer, migrasomes play a crucial role within the complex signaling network of the TME. They regulate the behavior of surrounding cells by transmitting various bioactive molecules, such as proteins, nucleic acids, and lipids, thereby influencing critical processes like angiogenesis, immune evasion, and metastasis [10]. For instance, Wu et al. [11] found that migrasome formation depends on the binding of integrins to extracellular matrix proteins, which is essential for effective cell migration—a key step in cancer metastasis. Additionally, migrasomes can enhance the immunosuppressive environment of tumors by carrying and releasing specific signaling molecules, promoting the infiltration and activation of immune cells such as T cells, macrophages, and dendritic cells (DCs) [12]. These functions highlight the significant role of migrasomes in modulating tumor biology and the immune microenvironment, potentially providing new targets and strategies for cancer therapy.

Research on migrasomes remains nascent, with their precise functions in lung adenocarcinoma (LUAD) largely uncharacterized. To address this knowledge gap, our study seeks to delineate the expression profiles and prognostic value of migrasome‐related genes (MIGgenes) in LUAD by amalgamating single‐cell and bulk transcriptomic datasets. Leveraging advanced machine learning algorithms, we have constructed a robust migrasome‐related signature (MIGsig) designed to forecast clinical outcomes and immunotherapy responses in LUAD patients.

## 2. Materials and Methods

### 2.1. Data Collection and Processing

We acquired gene expression data and corresponding clinical information for 541 LUAD tissue samples and 59 normal lung tissue samples from The Cancer Genome Atlas (TCGA) database. RNA‐seq gene expression data and clinical data were downloaded through the TCGA Genomic Data Commons (GDC) Data Portal. We performed initial quality control (QC) by first removing genes that were not expressed (count = 0) in any sample. We then applied a low‐count filter, retaining only genes that had a maximum expression value greater than 10 across the dataset. Expression data were normalized to eliminate technical variations. The external validation cohort′s data were sourced from the Gene Expression Omnibus (GEO) database, specifically the GSE50081, GSE31210, and GSE72094 datasets. These datasets include gene expression data and clinical information for LUAD patients. Background correction and normalization were performed on the data using the R package “limma.” The selection of MIGgenes was based on previously published studies [13], resulting in a total of 10 genes being included (Table S1).

To assess the risk model′s predictive power for immunotherapy efficacy, we analyzed three independent cohorts of immunotherapy‐treated patients. These included NSCLC patients receiving anti‐PD‐1/PD‐L1 therapy (GSE135222), melanoma patients managed with combined anti‐CTLA4 and anti‐PD1 therapy (GSE91061), and the IMvigor210 cohort of advanced urothelial cancer patients treated with the anti‐PD‐L1 agent atezolizumab.

### 2.2. Mutation and Copy Number Variation (CNV) Data

Regarding genomic alterations, the somatic mutation profiles (MAF format) were obtained from TCGA. Additionally, the corresponding CNV data for the LUAD cohort was retrieved from the UCSC Xena database.

### 2.3. Single‐Cell RNA‐seq Analysis

Single‐cell RNA sequencing data from seven early LUAD patients were collected from the GSE156632 dataset in the GEO database. QC was performed using the “Seurat” package, retaining cells with less than 10% mitochondrial gene content and genes expressed within a range of 200–7000. We first integrated the datasets and mitigated batch effects by applying the “Harmony” package. Subsequently, cell clusters were identified through the “FindNeighbors” and “FindClusters” functions, and the resulting community structure was visualized by t‐SNE embedding.

Gene set activity at the single‐cell level was quantified using Seurat′s “AddModuleScore” function. Subsequently, the “FindMarkers” function, also within Seurat, was applied to identify differentially expressed genes (DEGs) between defined cohorts, with statistical significance determined by the Wilcoxon test. Genes significantly altered between high and low migrasome‐scoring cells were implicated in migrasome‐related processes.

### 2.4. Single‐Sample Gene Set Enrichment Analysis (ssGSEA) and Gene Set Enrichment Analysis (GSEA)

Using the ssGSEA algorithm via the “GSVA” R package, we computed migrasome scores (MIGscores) for each TCGA‐LUAD sample. To explore signature‐related pathways, GSVA scores were profiled across 50 hallmark gene sets, with significantly differential pathways between risk groups identified using the “limma” package. Furthermore, GSEA was conducted on Gene Ontology (GO) gene sets (c5.go.v7.5.1.symbols.gmt) to elucidate the specific biological processes (BPs), cellular components (CCs), and molecular functions (MFs) enriched in different risk subgroups.

### 2.5. Weighted Gene Coexpression Network Analysis (WGCNA)

A weighted gene coexpression network was constructed from TCGA‐LUAD bulk RNA‐seq data using the R package “WGCNA.” After determining a suitable soft threshold (*β*) to ensure scale‐free topology, the weighted adjacency matrix was converted into a topological overlap matrix (TOM), from which a dissimilarity matrix (dissTOM) was derived. Gene modules were then identified through dynamic tree cutting. The module that demonstrated the strongest correlation with the MIGscore was selected for downstream analysis.

### 2.6. Construction of Prognostic Signature by Integrative Machine Learning Approaches

In the analysis of bulk RNA‐seq data from TCGA, we performed differential analysis to identify gene expression differences between normal and tumor samples. This analysis was conducted using the R package “limma,” with criteria set at |logFC| > 0.5 and *p*.*a* *d* *j* < 0.05. Subsequently, the DEGs unearthed from the bulk RNA‐seq investigation were intersected with the genes of the migrasome‐associated module ascertained through WGCNA. The obtained intersection gene is considered to be a candidate gene involved in the migration process, which is called MIGgenes.

MIGgenes with potential prognostic significance in the TCGA‐LUAD dataset were initially screened by univariate Cox regression. The cohort was then randomly divided into training and internal validation sets at a 1:1 ratio. Subsequently, we applied 10 machine learning algorithms to construct prognostic models, including Lasso, Ridge, stepwise Cox, CoxBoost, RSF, Enet, plsRcox, SuperPC, GBM, and survival‐SVM [14]. These algorithms generated 101 unique combinations, all trained on the training set using 10‐fold cross‐validation to optimize variable selection and construct prediction models. The performance of each model was initially assessed in the training set using the *C* index to evaluate its predictive accuracy. The top‐performing models based on the highest mean *C* index were then tested in the internal validation set to ensure their robustness and avoid overfitting. We excluded models that exhibited substantial performance drops in the internal validation set, focusing on models that maintained robust performance. The final selected model was then validated on external cohorts (GSE50081) to confirm its generalizability and prognostic utility. This step was purely for validation and not for model selection.

### 2.7. Survival Analysis and Construction of a Predictive Nomogram

Patients in the TCGA training, internal validation, and GSE50081 sets were stratified into high‐ and low‐risk groups based on the median MIGsig risk score. Kaplan–Meier analysis, executed with the “survminer” R package (log‐rank test, *p* < 0.05), was used to compare significant disparities in overall survival (OS), progression‐free survival (PFS), and disease‐specific survival (DSS) between these groups [15]. The predictive accuracy of MIGsig for OS was evaluated by receiver operating characteristic (ROC) analysis using the “timeROC” package. Furthermore, the area under the curve (AUC) of MIGsig was compared against that of other clinical characteristics to assess its relative prognostic strength [15]. To enhance the prognostic precision and predictive efficacy of our model, we formulated a nomogram incorporating MIGsig alongside relevant clinical variables. We assessed the performance of the nomogram by evaluating its precision, discrimination, accuracy, and net clinical benefit using ROC curves, the *C* index, calibration curves, and decision curve analysis (DCA).

### 2.8. Comprehensive Analysis of Immune‐Omics Molecular Characterization and Immunotherapy Response Based on MIGsig

We utilized the Immuno‐Oncology Biological Research (IOBR) R package to gather a comprehensive collection of previously documented signatures related to different aspects of the TME, such as cell types, immunotherapy responses, immune suppression, and immune exclusion. We systematically evaluated the tumor immune microenvironment by quantifying enrichment scores for individual samples, enabling a comparative analysis of immunological landscapes between MIGsig‐defined high‐ and low‐expression cohorts. Differences in tumor mutational burden (TMB), tumor neoantigen burden (TNB), and fibroblast infiltration were assessed between these groups. Patients were then stratified based on the MIGsig for subsequent immunotherapy response prediction. This predictive profiling integrated the tracking tumor immunophenotype (TIP) algorithm, subclass mapping, and the tumor immune dysfunction and exclusion (TIDE) framework to estimate the likelihood of a favorable immunotherapy response, with a focus on patients demonstrating delayed clinical benefit. All findings were robustly validated in independent immunotherapy cohorts (GSE135222 and GSE91061).

### 2.9. In Silico Analysis to Screen Potential Therapy Agents for Patients With High MIGsig

Drug sensitivity data for human cancer cell lines (CCLs) was sourced from the CTRP v.2.0 and PRISM Repurposing databases. The responsiveness of CCLs to specific compounds was quantified using the area under the dose–response curve (AUC), which served as the primary metric for drug sensitivity.

### 2.10. Tissue Sample Collection and Cell Line Culture

Approved by the Ethical Committee of The Second Hospital of Hebei Medical University (2025‐Y173), this study utilized 20 pairs of snap‐frozen LUAD tissues and matched adjacent normal samples. These specimens were obtained from initial surgical resections between June and July 2024, with informed consent secured from all participating patients.

The LUAD cell lines (A549, H1299, and HCC827) and the normal bronchial epithelial cell line BEAS2B were procured from the Cell Repository of the Chinese Academy of Sciences (Shanghai, China). All cells were cultured in RPMI‐1640 medium containing 10% fetal bovine serum (FBS) at 37°C in a humidified 5% CO_2_ atmosphere.

### 2.11. Quantitative Real‐Time Polymerase Chain Reaction (qRT‐PCR)

Total RNA was isolated from cells with TRIzol reagent (Invitrogen) and subsequently reverse‐transcribed into cDNA using the PrimeScript RT kit (Takara). qRT‐PCR was then performed on a CFX96 system (Bio‐Rad) with SYBR Premix Ex Taq (Takara). Gene expression levels were quantified relative to the GAPDH internal control via the 2^-*ΔΔ*Ct^ method. All primers, sequences of which are provided in Table S2, were synthesized by Tsingke Biotech.

### 2.12. Statistical Analysis

The statistical analyses in this study were performed utilizing R software, specifically Version 4.0.1, as previously indicated. A significance threshold of *p* < 0.05 was employed to ascertain statistical significance.

## 3. Results

### 3.1. Migrasome Characteristic in Single‐Cell Transcriptome

Single‐cell RNA sequencing data was obtained from seven individuals diagnosed with early‐stage LUAD, comprising a total of 10,999 cells. Using marker genes specific to different cell types, the cellular composition was identified and classified into eight predominant clusters: T cells, macrophages, NK cells, epithelial cells, B cells, mast cells, fibroblasts, and endothelial cells (Figure 1a). The heat map in Figure 1b displays the top four marker genes associated with each identified cell population.

Figure 1Migrasomes characteristic in the single cell transcriptome. (a) The t‐SNE plot displays the identified cell types based on marker genes. (b) The heat map illustrates the Top 4 marker genes in each cell cluster. (c) The activity score of migrasomes is depicted for each cell. (d) The distribution of the migrasomes score is analyzed across different cell types.

**Figure:**
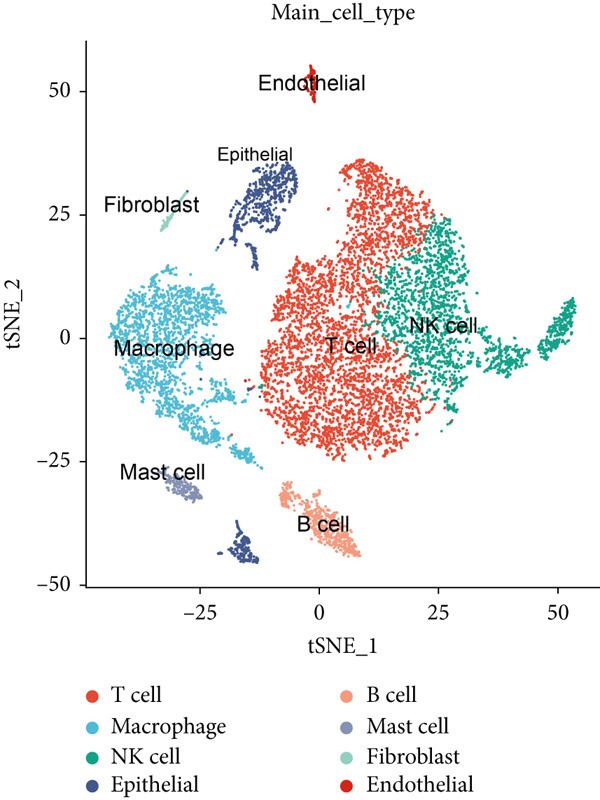
(a)

**Figure:**
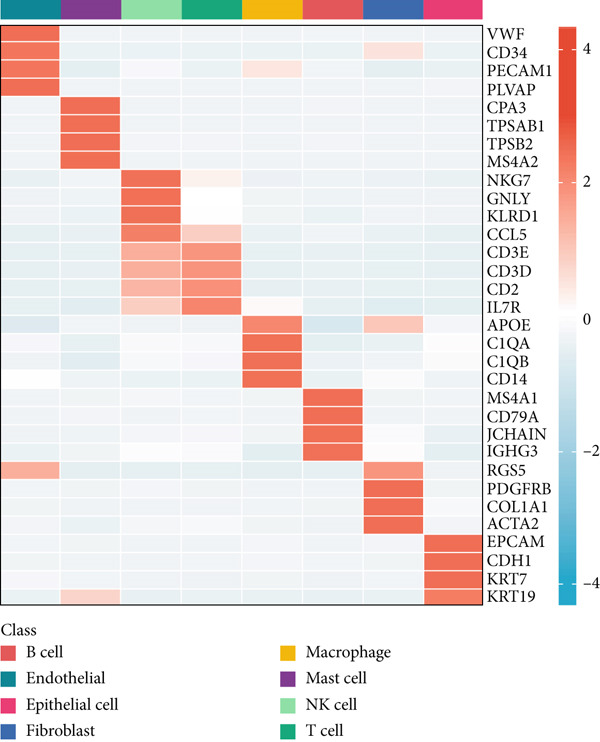
(b)

**Figure:**
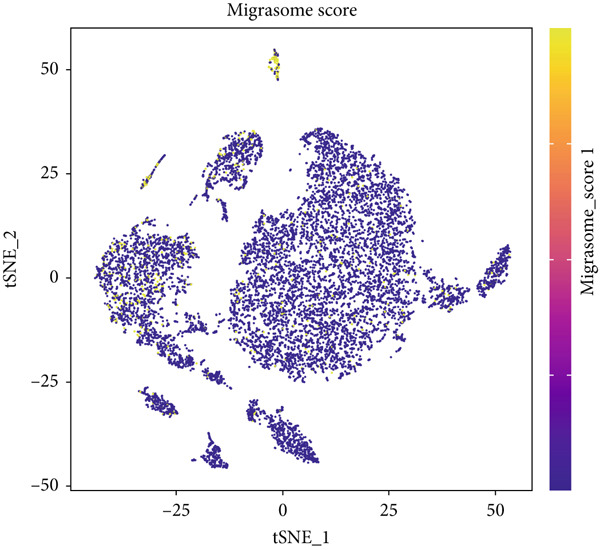
(c)

**Figure:**
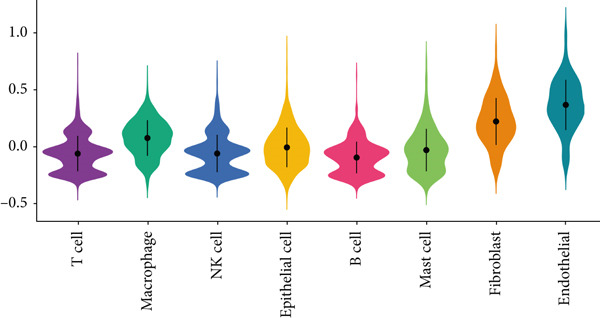
(d)

To evaluate migrasomes activity in different cell types, we utilized the “AddModuleScore” function in the Seurat package to calculate the expression levels of a curated 10‐gene set associated with migrasomes functionality across the entire cell population (Figure 1c). Notably, among the eight distinct cell types investigated, we observed a significant increase in migrasomes activity specifically in endothelial cells and fibroblasts (Figure 1d). Leveraging this migrasomes′ activity metric, we stratified cells into high‐ and low‐migrasome categories, ′facilitating the identification of 2969 DEGs between these two groups for subsequent in‐depth analysis.

### 3.2. Identification of the Hub Module and Genes Related to Migrasomes in Bulk RNA‐seq

In this investigation, we employed the ssGSEA algorithm to compute the migrasomes activity score concerning individual samples sourced from the TCGA‐LUAD dataset. To identify modules that were significantly correlated with the MIGscore, we conducted a WGCNA analysis and assigned the 2969 DEGs associated with migrasomes, as identified at the single‐cell RNA sequencing level, to similar modules based on their expression patterns. This analysis resulted in the identification of 10 modules (Figure 2a). The results of our investigation revealed a robust correlation between the MEmagenta module and MIGscores in bulk RNA‐seq (cor = 0.65, Figure 2b). A strong positive correlation (cor = 0.79) was observed in the scatter plot of gene significance (GS) versus module membership (MM) for the magenta module (Figure 2c), implying that its constituent genes are functionally significant in migrasome‐related processes.

Figure 2Identification of the MIGgenes. (a) The cluster dendrogram illustrates the results from the WGCNA analysis. (b) The module–trait heat map demonstrates the close relationship between the MEmagenta module and the migrasome trait. (c) The scatter plot reveals the correlation between gene significance (GS) and module membership (MM) within the magenta module. (d) The volcano plot displays the results of the differential analysis comparing TCGA‐LUAD tumor samples and normal samples. (e) The Venn plot highlights the intersecting genes between the MEmagenta module and DEGs identified in bulk RNA‐seq. (f) The GO enrichment analysis reveals the functional enrichment of the MIGgenes. (g) Results of the univariate Cox regression analysis for MIGgenes and the correlations among these genes (circle size represents the p‐value). (h) Copy number variation (CNV) frequency of MIGgenes.

**Figure:**
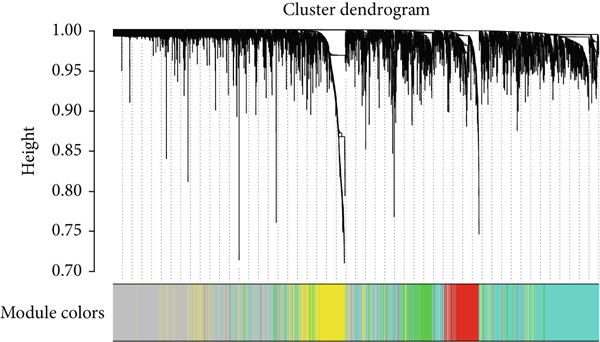
(a)

**Figure:**
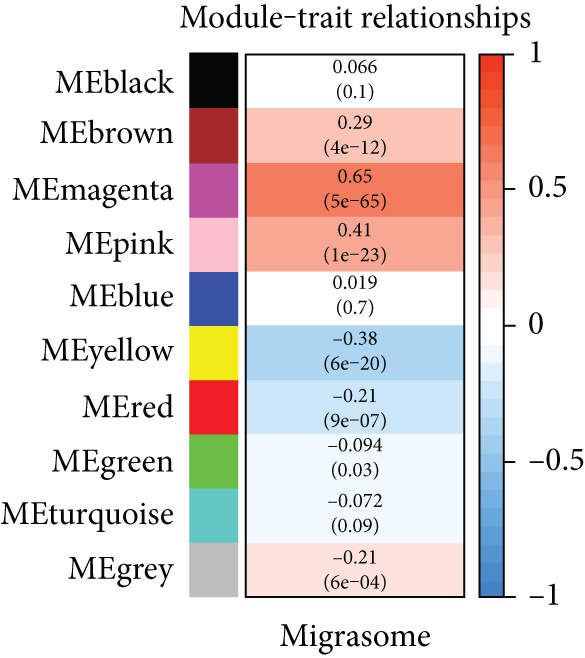
(b)

**Figure:**
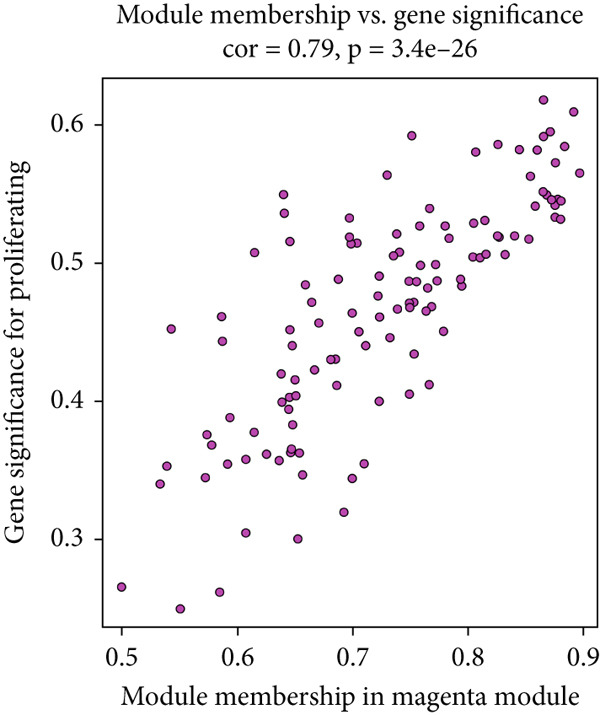
(c)

**Figure:**
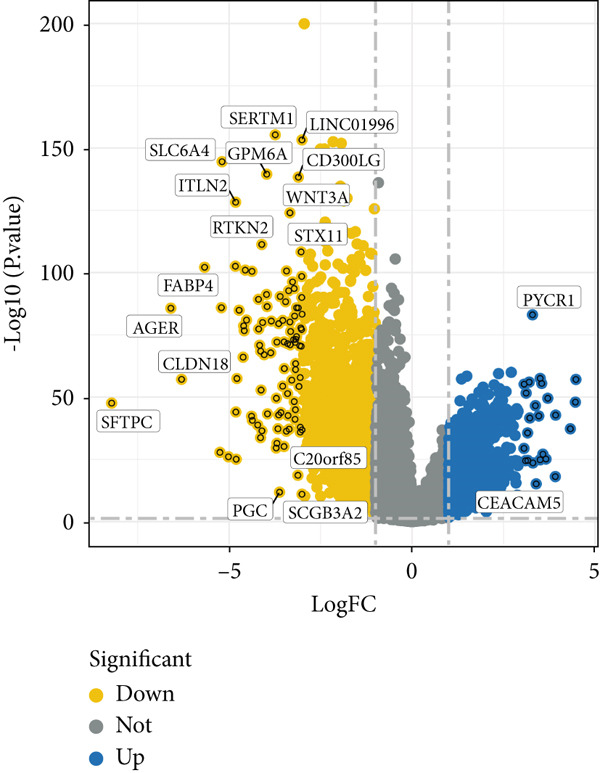
(d)

**Figure:**
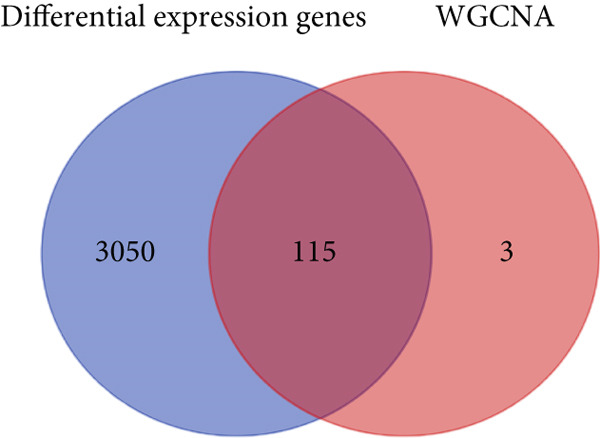
(e)

**Figure:**
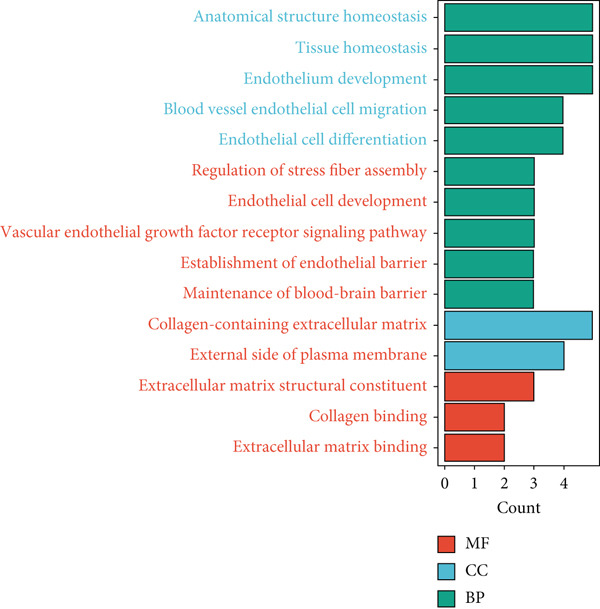
(f)

**Figure:**
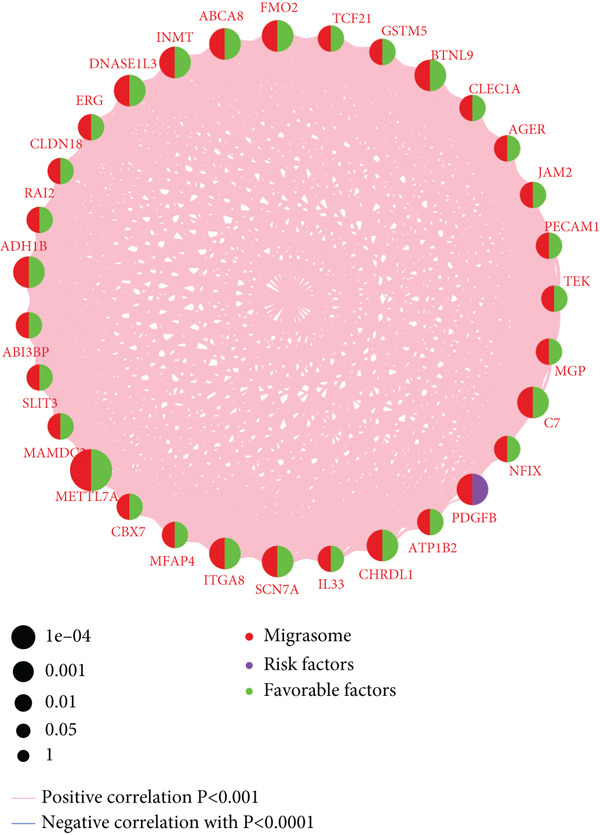
(g)

**Figure:**
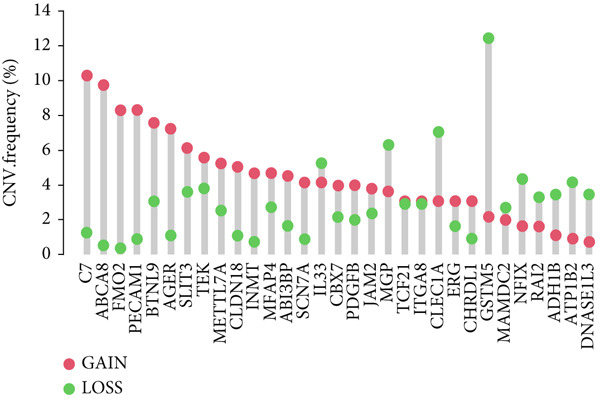
(h)

The volcano plot (Figure 2d) illustrates the DEGs identified between tumor and normal lung tissues in the TCGA‐LUAD bulk RNA‐seq dataset. The selection criteria for these DEGs were |logFC| > 0.5 and *p*.adj < 0.05. Simultaneously, the intersection of the 118 genes within the magenta module with the aforementioned DEGs from the bulk RNA‐seq analysis resulted in a subset of 115 genes (Figure 2e). The MIGgene subset demonstrated prominent involvement in migrasome dynamics across both bulk and single‐cell transcriptomic landscapes. GO enrichment analysis revealed significant associations with key BPs, including collagen/extracellular matrix binding, plasma membrane architecture, and endothelial development pathways (Figure 2f). Subsequent univariate Cox regression of the 115 MIGgenes identified 31 genes with significant prognostic value (*p* < 0.05).

### 3.3. Construction of a Prognosis Signature Based on Integrative Machine Learning

In the single‐cell and bulk transcriptome analyses, a total of 115 MIGgenes were identified, with 31 of them showing significant prognostic associations (adjusted *p* value < 0.05, using the Benjamini–Hochberg method). These genes were used to construct the MIGsig within the machine learning framework. Among the 101 assessed models, the foremost five predictive models, as determined by the mean *C* index, were formulated utilizing the RSF algorithm. These models, however, exhibited substantial performance drops in the internal validation set, indicating potential overfitting. Consequently, we selected the Lasso + StepCox combined model, which achieved the highest average *C* index of 0.703 in the training set and maintained robust performance in the internal validation set (Figure 3a).

Figure 3A consensus MIGsig model was developed and validated via the machine learning–based integrative procedure. (a) The distribution of the *C* index for all 101 models in a 10‐fold cross‐validation framework. The Lasso–Cox model (marked in red) ranked sixth but demonstrated stable *C* index performance. (b) Visualization of Lasso regression analysis, showing the optimal *λ* value determined by minimizing the partial likelihood deviance. (c) Regression coefficients of genes obtained from Lasso regression analysis and results from stepwise Cox regression analysis. (d) The risk score calculated for each patient using the Lasso–Cox model, dividing patients into the high‐risk and low‐risk groups based on the median risk score. (e) The forest plots depict the results of stepwise Cox regression analysis. (f) The distribution of the risk score and overall survival status of patients in the TCGA‐LUAD cohort are illustrated. (g–k) KM curve analysis comparing overall survival (OS), progression‐free survival (PFS), and disease‐specific survival (DSS) between the high‐risk and low‐risk groups in the training set, internal validation set, and external validation set.

**Figure:**
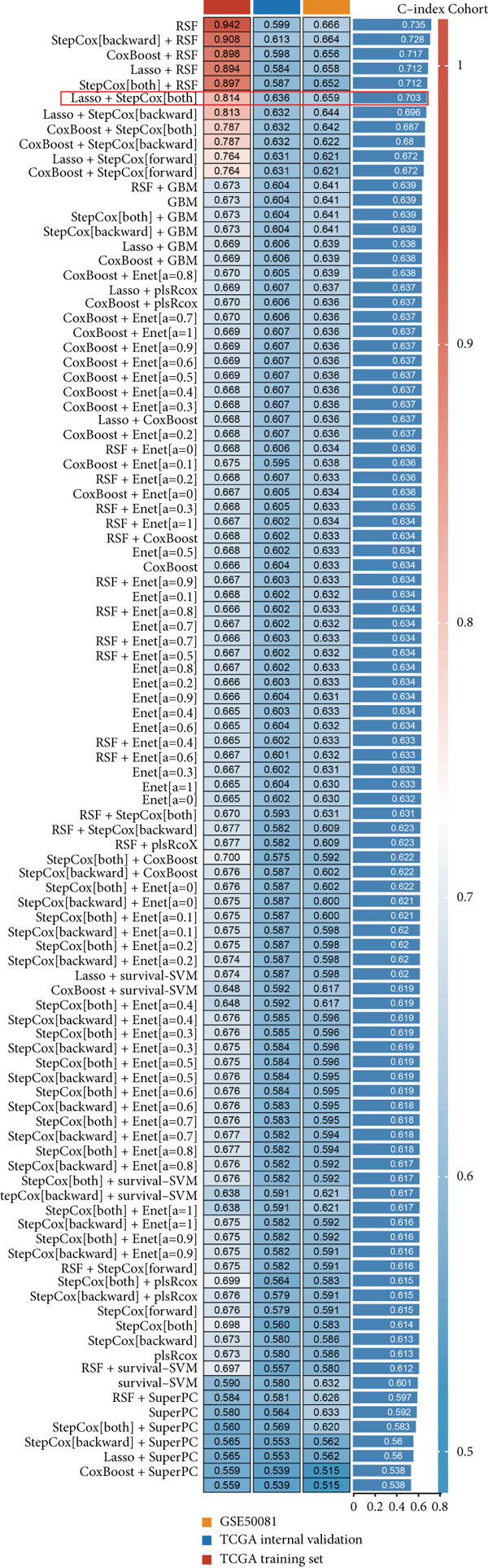
(a)

**Figure:**
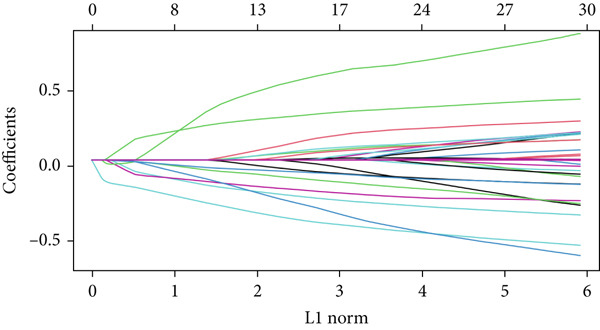
(b)

**Figure:**
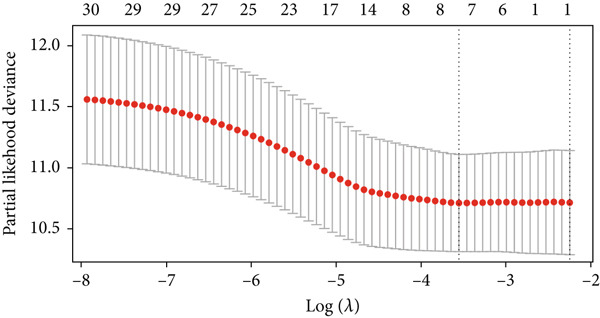
(c)

**Figure:**
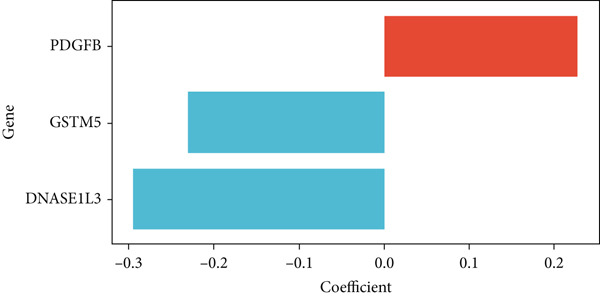
(d)

**Figure:**
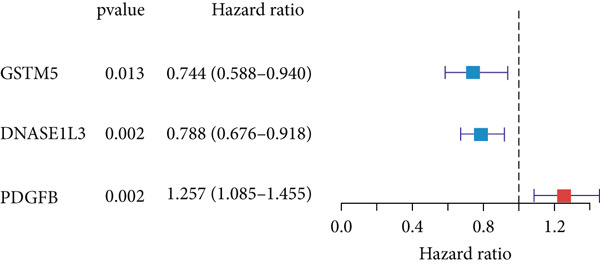
(e)

**Figure:**
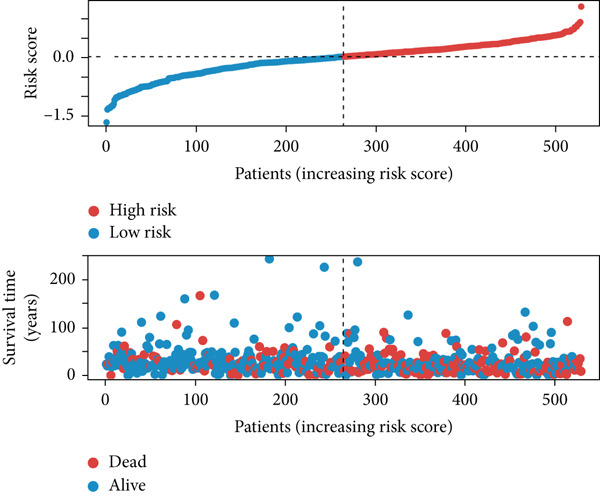
(f)

**Figure:**
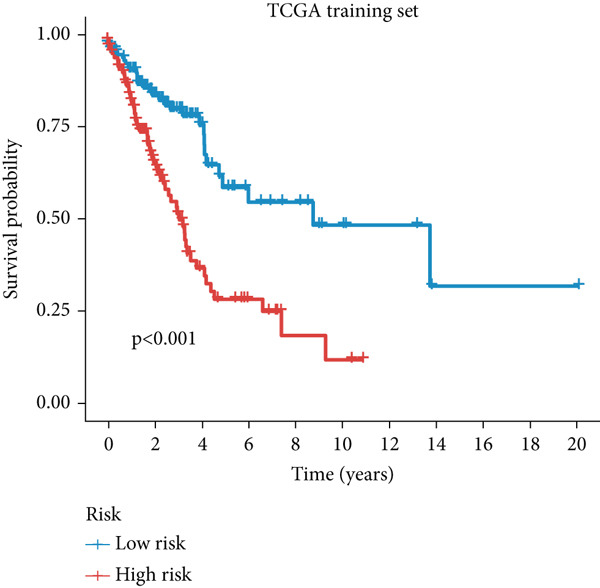
(g)

**Figure:**
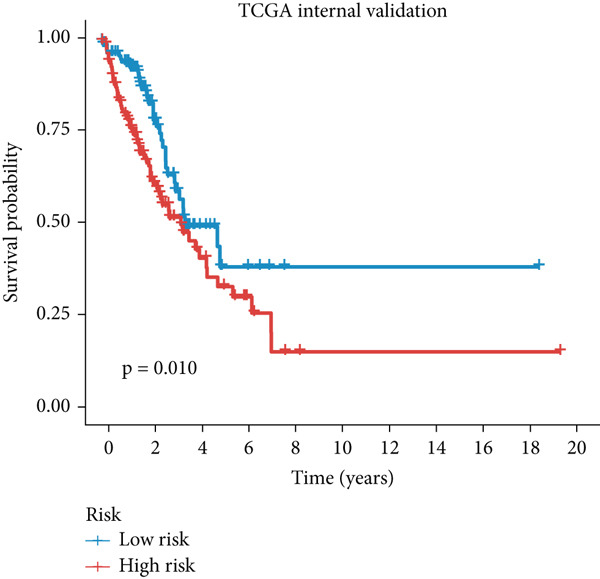
(h)

**Figure:**
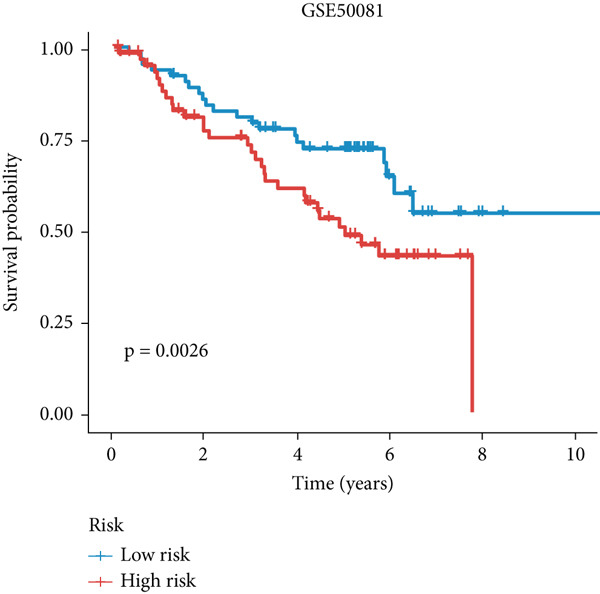
(i)

**Figure:**
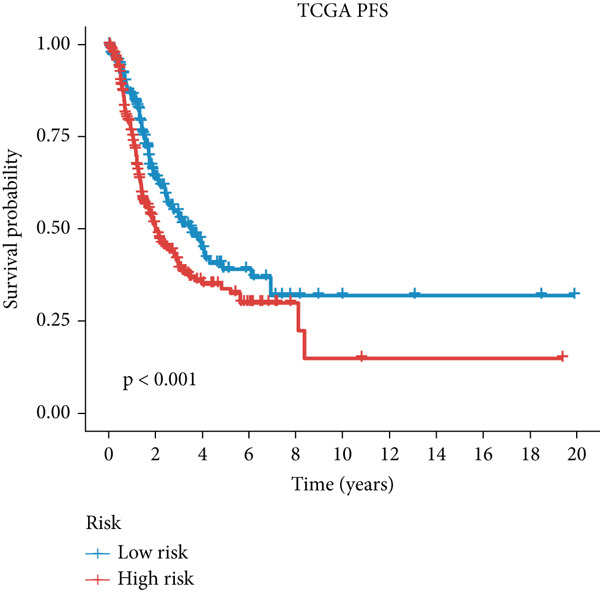
(j)

**Figure:**
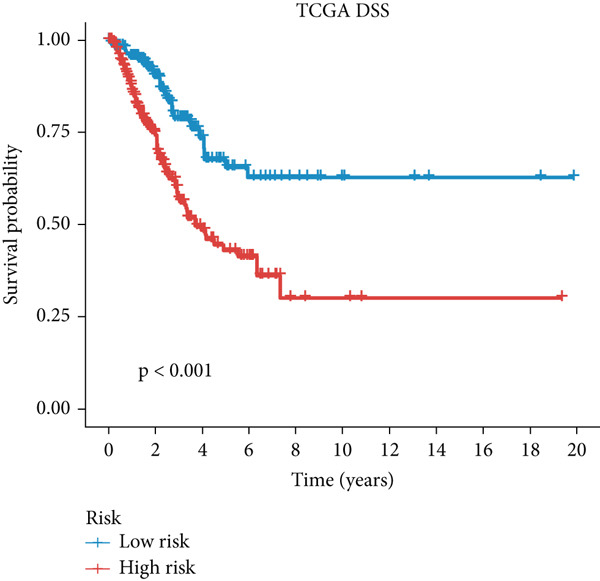
(k)

Employing a 10‐fold cross‐validation approach, the optimal *λ* value of 0.03021431 was ascertained via LASSO analysis with the objective of minimizing partial likelihood deviation (Figure 3b,c). Subsequently, genes exhibiting nonzero coefficients in the LASSO analysis underwent stepwise Cox proportional hazards regression analysis. From this analysis, we identified a set of three genes (Figure 3d). Following calculation, we derived individual risk scores for each patient through a weighting of the expression levels of the three genes, employing regression coefficients acquired from the Cox model analysis (Figure 3e). Subsequently, patients were stratified into distinct risk groups, namely, high‐risk and low‐risk, based on the median risk score. Noteworthy is the observed correlation between escalating risk scores and an associated rise in mortality incidences among patients (Figure 3f). Moreover, across the training set, internal validation set, and GSE50081 dataset, individuals classified into the high‐risk category demonstrated notably diminished OS outcomes in contrast to their counterparts categorized as low‐risk (Figures 3g, 3h, and 3i). Correspondingly, those individuals identified as low‐risk exhibited markedly superior PFS and DSS rates compared to their high‐risk counterparts (Figure 3j,k).

### 3.4. The Predictive Accuracy and Clinical Translational Significance of MIGsig

In the training set, the AUC of the MIGsig was 0.741, 0.682, and 0.591 for the 1‐, 3‐, and 5‐year intervals, respectively (Figure 4a). In the internal validation set, AUC values of 0.680, 0.559, and 0.626 were observed (Figure 4b), while in the GSE50081 dataset, AUC values of 0.672, 0.678, and 0.633 were recorded (Figure 4c). Furthermore, comparative analyses were conducted between the AUC of MIGsig and various clinical parameters such as age, gender, stage, grade, and T. Remarkably, the AUC of the MIGsig demonstrated significant superiority over these clinical characteristics (Figures 4d, 4e, and 4f).

Figure 4Evaluation of the MIGsig. (a–c) The ROC curves are presented to demonstrate the specificity and sensitivity of MIGsig in predicting 1‐, 3‐, and 5‐year OS in the TCGA training set (a), TCGA internal validation set (b), and GSE50081 dataset (c). (d–f) The ROC curves compare the predictive performance of MIGsig with clinical characteristics in the training set (d), internal validation set (e), and GSE50081 dataset (f). (g) The distribution of clinical characteristics and the expression of model genes is presented based on the MIGsig risk score. (h) The correlation between the MIGsig low‐ and high‐risk groups and clinical characteristics is examined. (i) The difference in risk score between patients is analyzed according to their stage classification. (j) The proportion of each stage is determined within the MIGsig risk subgroups. (k, l) The KM curves demonstrate the consistent performance of the MIGsig in different subgroups (based on stage) of LUAD patients.

**Figure:**
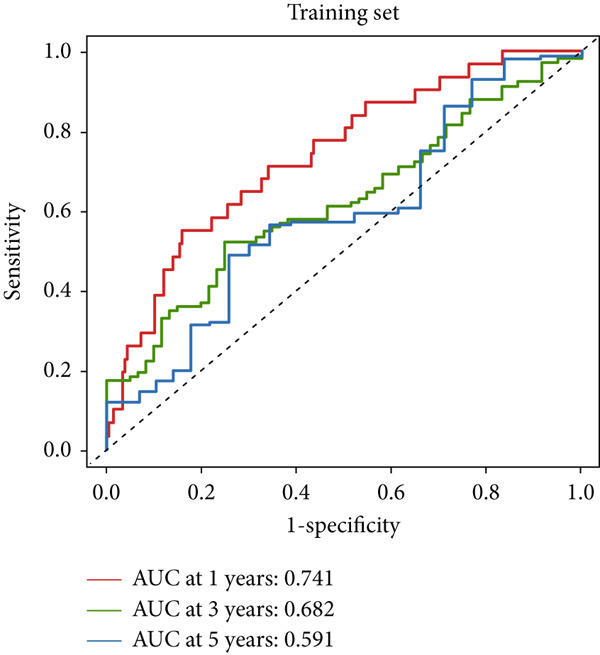
(a)

**Figure:**
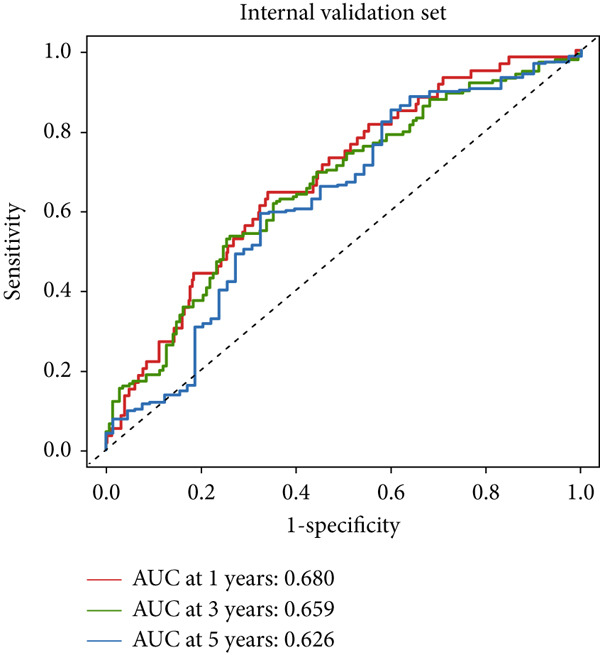
(b)

**Figure:**
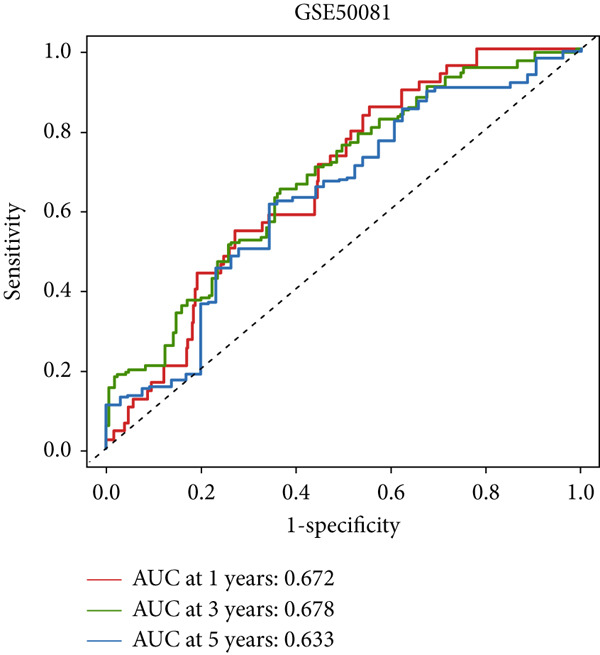
(c)

**Figure:**
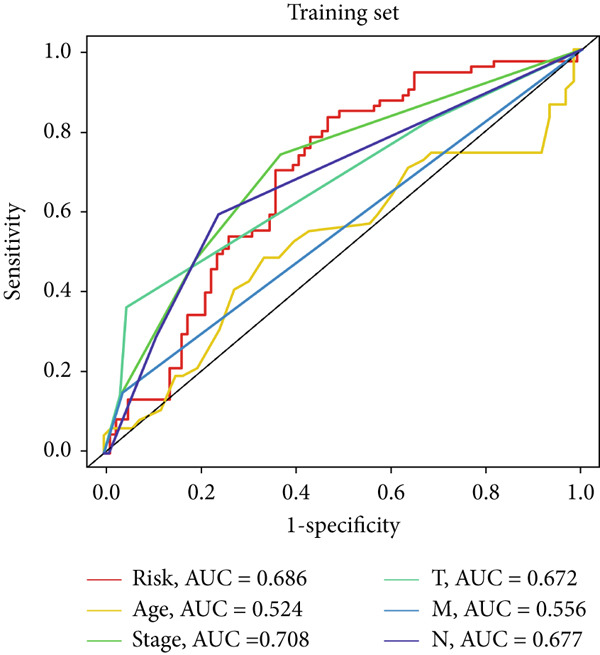
(d)

**Figure:**
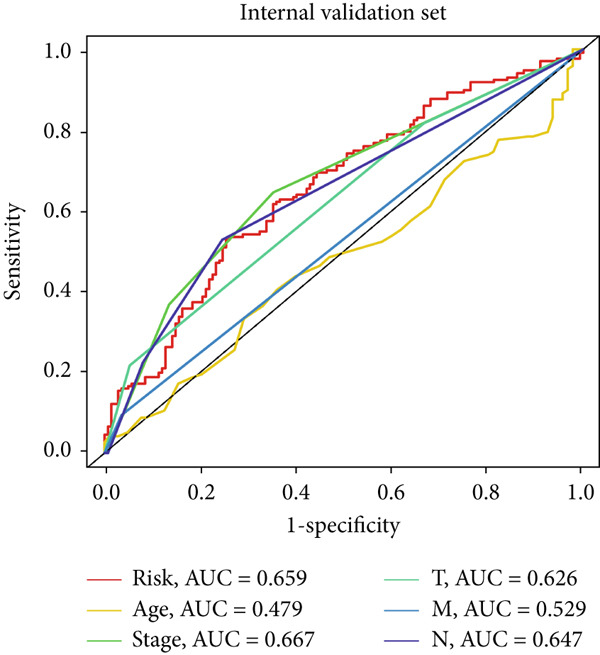
(e)

**Figure:**
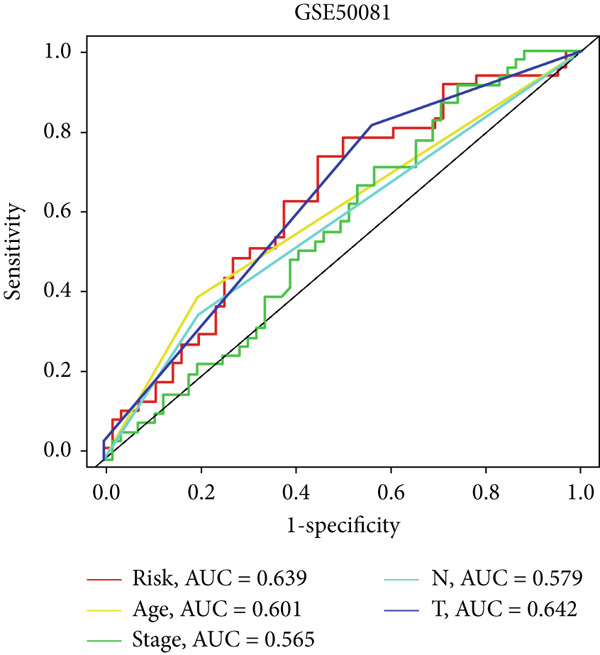
(f)

**Figure:**
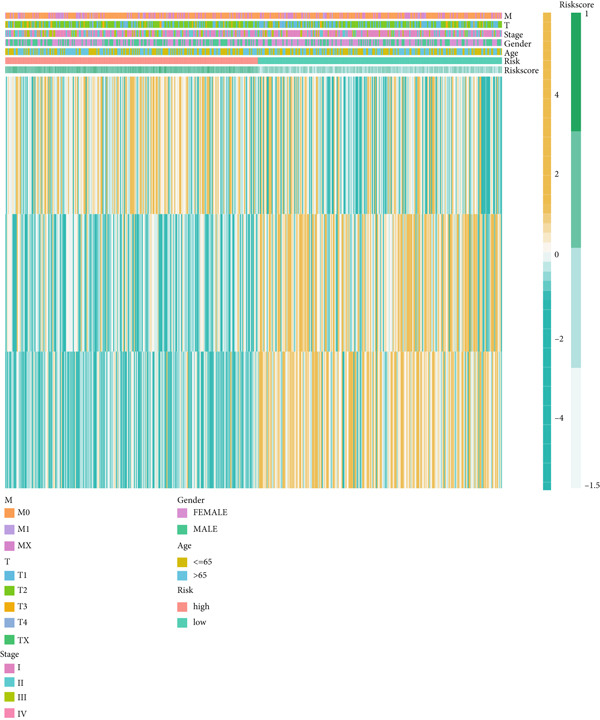
(g)

**Figure:**
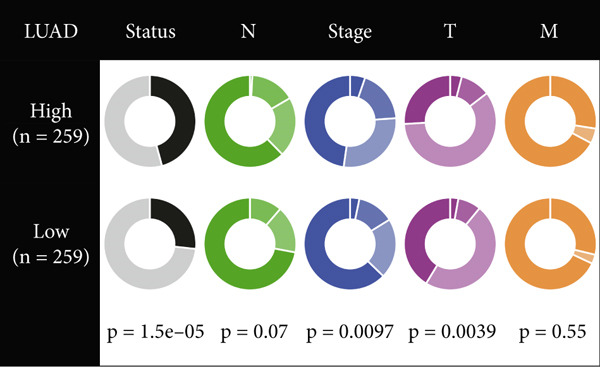
(h)

**Figure:**
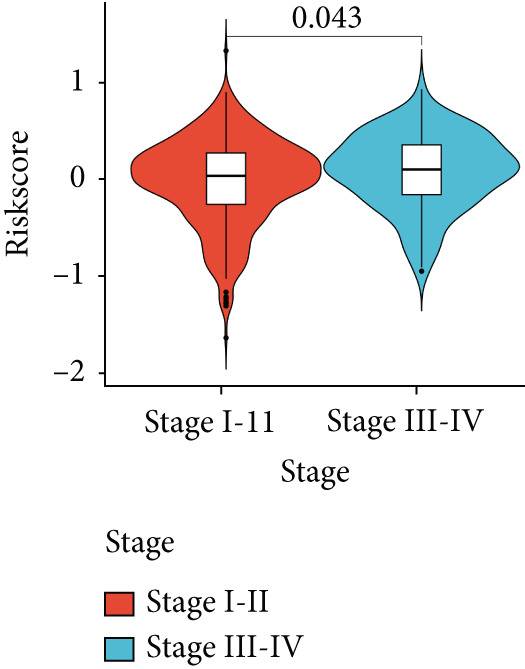
(i)

**Figure:**
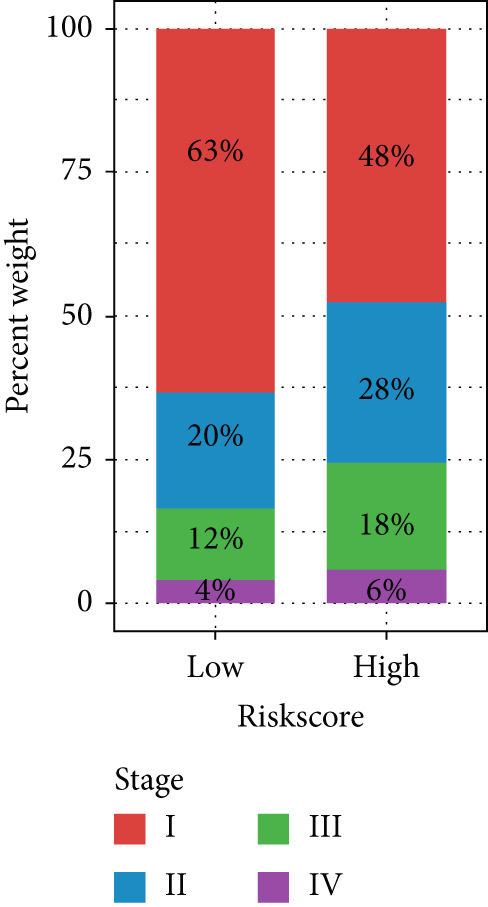
(j)

**Figure:**
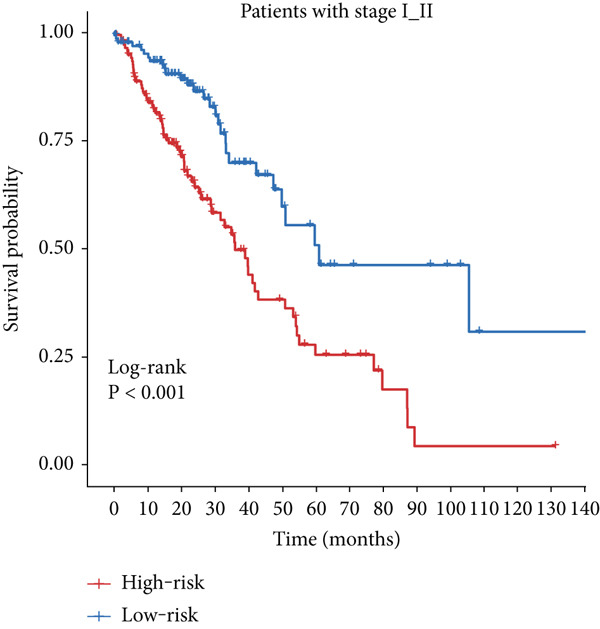
(k)

**Figure:**
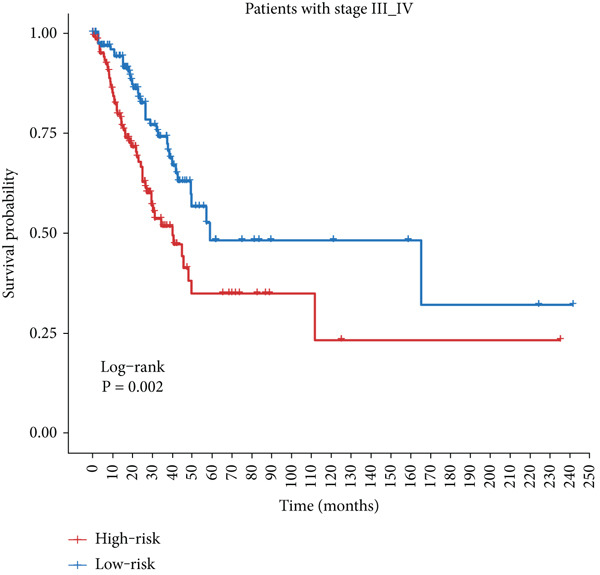
(l)

In the assessment of prognosis for patients with LUAD, clinical characteristics play a pivotal role in clinical practice. To ascertain the relationship between the MIGsig and diverse clinical parameters, we conducted a comprehensive analysis. Utilizing the TCGA‐LUAD dataset, we discerned noteworthy variations in the distribution of grade, stage, T, and M statuses between cohorts classified as high and low risk (*p* < 0.001, Chi‐squared test) (Figure 4g,h). Moreover, our observations revealed a notable elevation in risk scores among patients at Stages III–IV in comparison to those at Stages I–II (Figure 4i). Additionally, a higher representation of patients in the intermediate and advanced stages was evident within the high‐risk group (Figure 4j). Using KM curve analysis, our study revealed a consistent prognostic ability of the MIGsig across subgroups classified by stage in the context of LUAD (Figure 4k,l). These findings indicate a clear association between the MIGsig and an unfavorable prognosis among patients diagnosed with LUAD.

### 3.5. Comparison of Prognostic Value Between MIGsig Signature Score and Clinical Features

The MIGsig demonstrated superior discriminative ability (*C* index) compared to standard clinical parameters including age, sex, and TNM stage (Figure 5a). We further benchmarked MIGsig against 22 previously published prognostic models across multiple datasets (TCGA training/validation sets, GSE31210, GSE72094, and GSE50081). As shown in Figure 5b, our model consistently achieved a higher *C* index in all these cohorts, confirming its robust predictive performance.

Figure 5Comparison of prognostic value between MIGsig signature score and clinical features. (a) Box plot of *C* index of MIGsig signature score and clinical indicators in various datasets. (b) Comparison of MIGsig signature score and other published prognostic models in various datasets.

**Figure:**
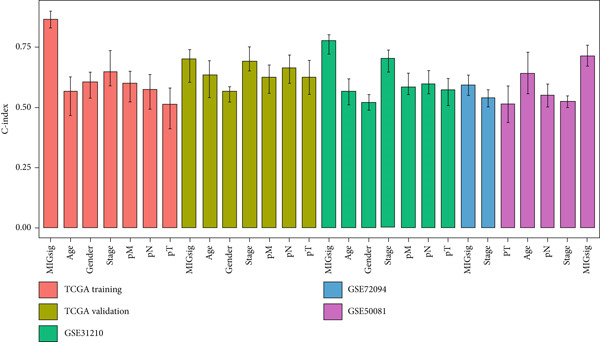
(a)

**Figure:**
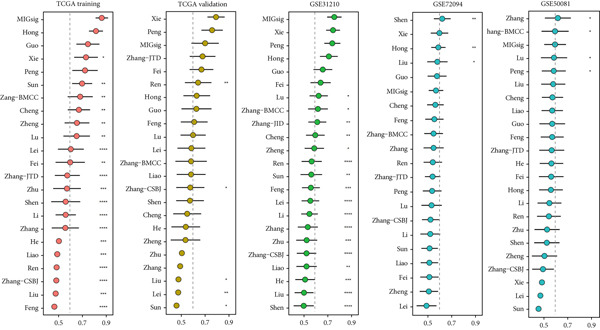
(b)

### 3.6. Establishment and Validation of a Nomogram Combined With Clinical Characteristics

To evaluate the independent prognostic value of MIGsig in LUAD, univariate and multivariate Cox regression analyses for OS, PFS, and DSS were conducted in the TCGA‐LUAD cohort. The MIGsig emerged as a significant risk factor for all three endpoints in univariate analysis and, critically, retained its independent prognostic power in multivariate analyses (Figures 6a, 6b, and 6c). This robust association was corroborated in the independent GSE50081 dataset (Figure 6d).

Figure 6Establishment and verification of the nomogram. (a–c) The TCGA‐LUAD cohort underwent univariate and multivariate analyses to assess the impact of clinical characteristics and MIGsig on OS (a), PFS (b), and DSS (c). (d) Similarly, univariate and multivariate analyses were performed on the GSE50081 dataset to evaluate the association between clinical characteristics, MIGsig, and OS. (e) A nomogram was constructed based on the MIGsig and clinical characteristics, such as age, stage, T, and N. (f) The calibration curve of the nomogram is plotted to assess its accuracy in predicting 1‐, 3‐, and 5‐year OS. (g) The ROC curves illustrate the prediction performance of the nomogram for 1‐, 3‐, and 5‐year OS. (h) The *C* index is compared between the nomogram and other clinical characteristics to evaluate their predictive abilities. (i) DCA is conducted to assess the net benefit of using the nomogram compared to other clinical characteristics.

**Figure:**
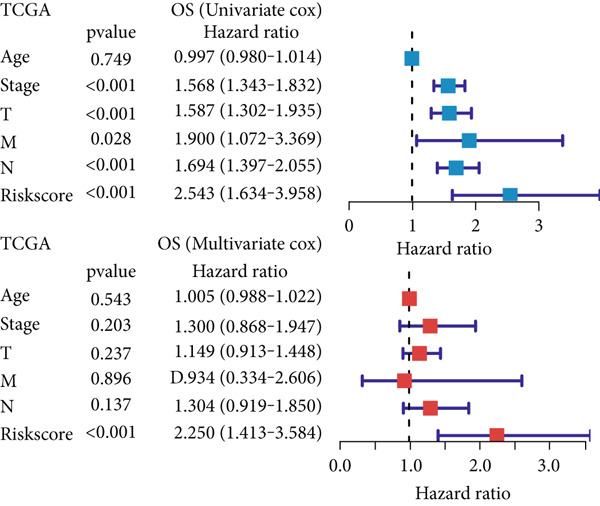
(a)

**Figure:**
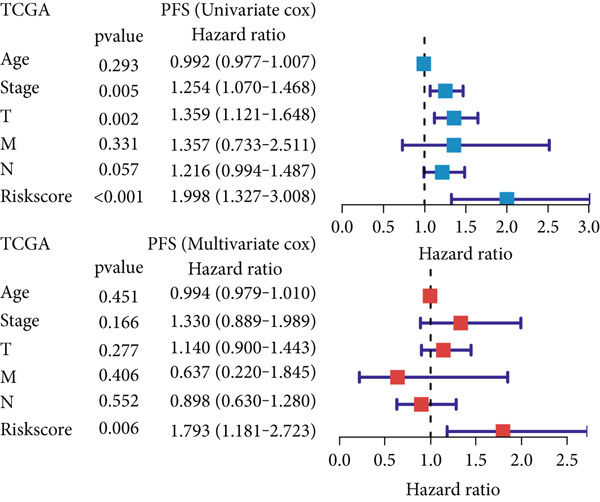
(b)

**Figure:**
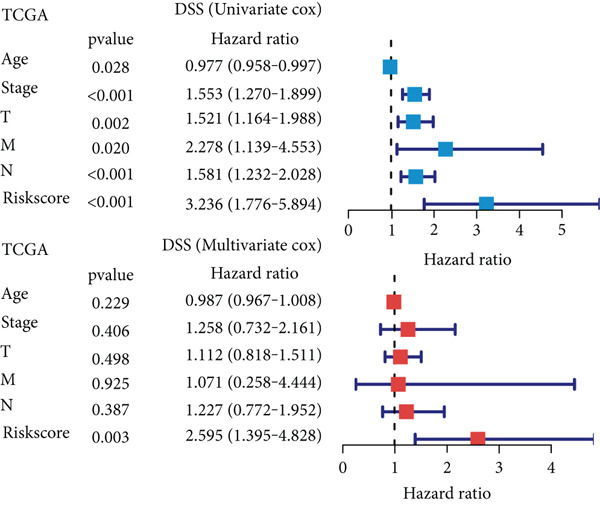
(c)

**Figure:**
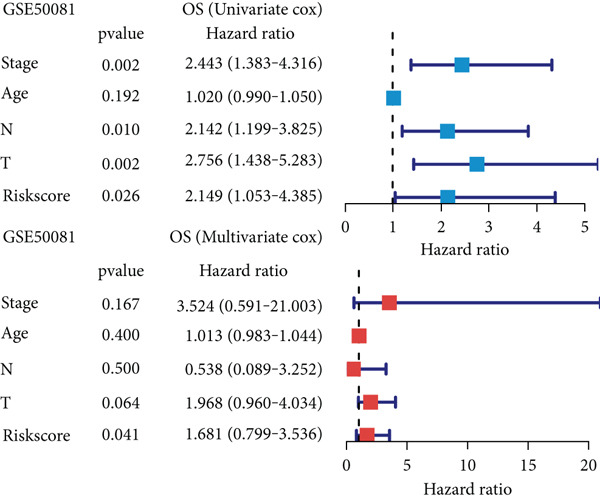
(d)

**Figure:**
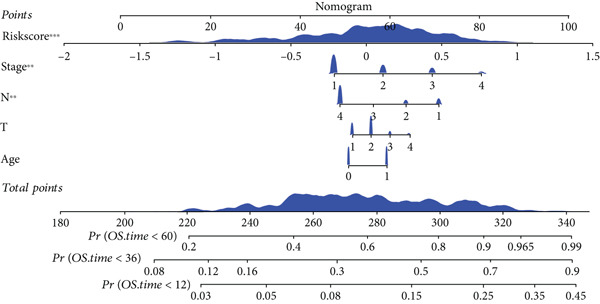
(e)

**Figure:**
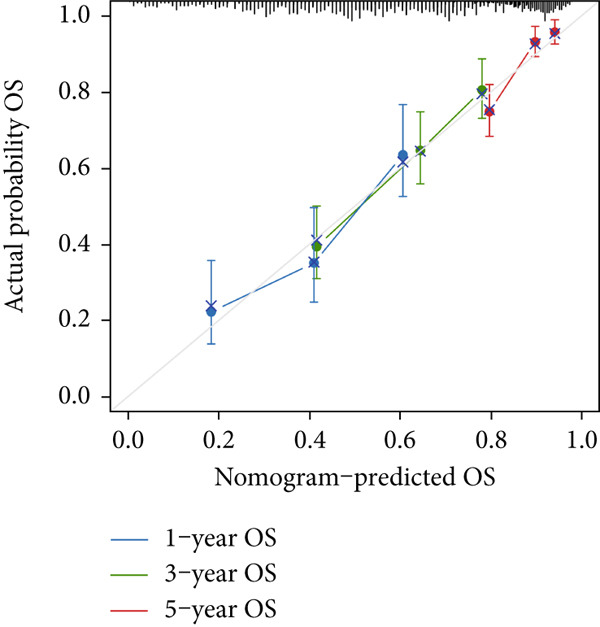
(f)

**Figure:**
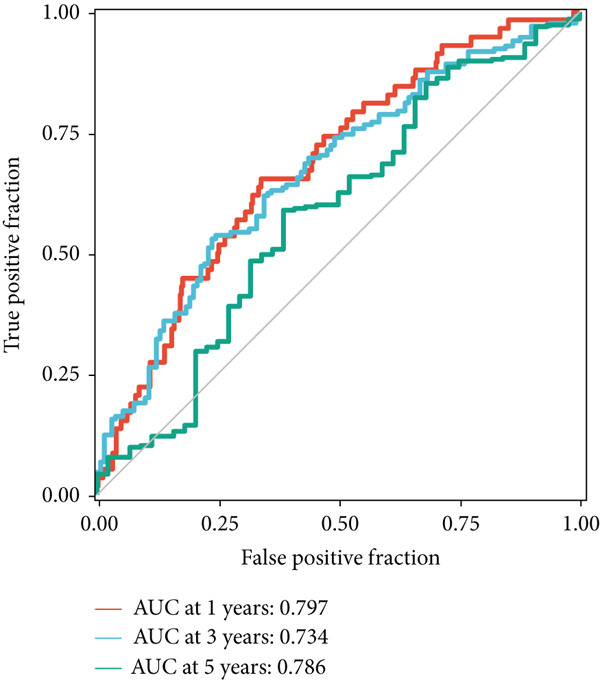
(g)

**Figure:**
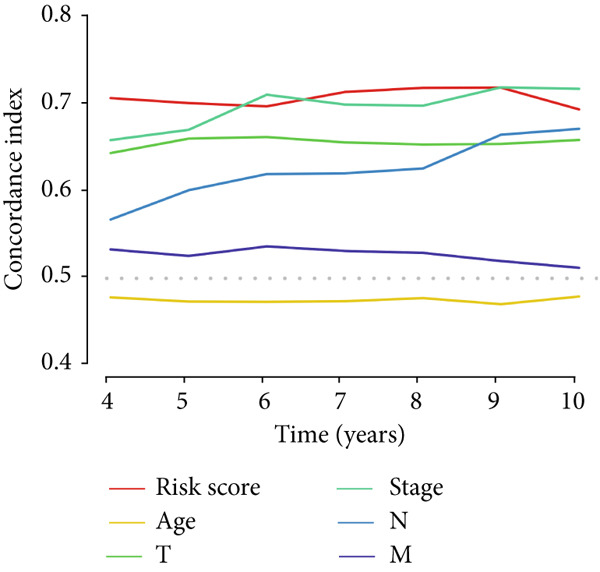
(h)

**Figure:**
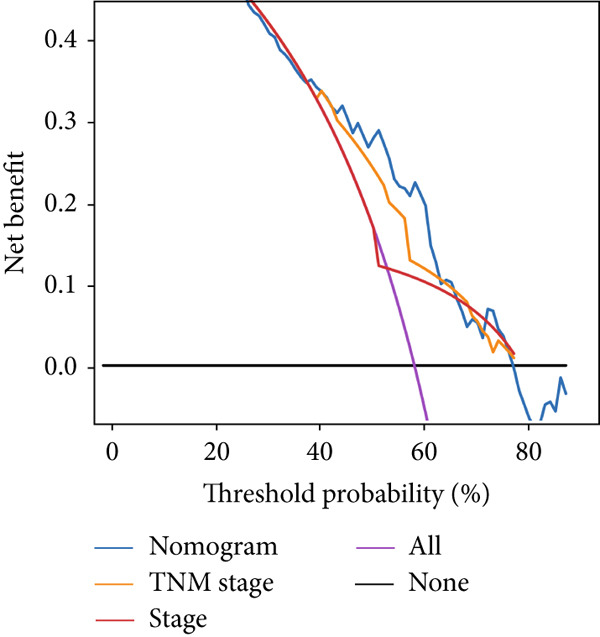
(i)

In the pursuit of enhancing the clinical applicability of MIGsig, a nomogram was formulated by integrating MIGsig data with pertinent clinical characteristics (Figure 6e). Evaluation of the nomogram′s performance through calibration curves revealed commendable concordance between the predicted outcomes and observed results (Figure 6f). Notably, the AUC for the nomogram exhibited substantial predictive accuracy at different temporal intervals—0.797, 0.734, and 0.786 for 1‐, 3‐, and 5‐year durations, respectively (Figure 6g). Moreover, the stability and robust predictive capacity of the nomogram were evidenced by the performance of the *C* index. This performance surpassed that of alternative clinical characteristics in forecasting OS across the 1–10‐year timeframe (Figure 6h). Notably, the DCA further underscored the superiority of the nomogram by illustrating a superior net clinical benefit compared to other clinical features (Figure 6i). Collectively, these observations substantiate the efficacy of the nomogram, constructed based on MIGsig, as a dependable and precise tool for individualized prognosis prediction in patients with LUAD.

### 3.7. Underlying Molecular Mechanisms of the MIGsig in Bulk Transcriptome

To delineate the molecular mechanisms linking MIGsig to LUAD prognosis, we performed functional enrichment analyses. GSEA revealed a conspicuous divergence between the risk groups: low‐MIGsig samples were enriched in vesicular transport processes (e.g., clathrin‐coated vesicle and transepithelial transport; Figure 7a), whereas high‐MIGsig samples showed enrichment in endosome‐related pathways (Figure 7c). GSVA further corroborated this functional split, identifying activated KRAS signaling in the high‐risk group, and heightened activity of PI3K/AKT/mTOR, E2F/MYC targets, and G2M checkpoint in the low‐risk group (Figure 7b). Correlation analysis between MIGsig expression and hallmark pathway scores further substantiated this observation (Figure 7d). These results underscore the robust linkage between MIGsig expression and pivotal cancer‐related BPs as well as metabolic pathways.

Figure 7The transcriptome features of patients with various MIGsig in LUAD. (a) The ridge plot visualizes the GO terms enriched in the low‐MIGsig group. (b) Differences in hallmark pathway activities between the high‐ and low‐MIGsig groups are measured and scored using the GSVA method. (c) The GO terms enriched in the high‐ and low‐MIGsig groups are determined through GSEA. (d) The correlation between the MIGsig and hallmark pathway activities, scored by GSVA, is examined.

**Figure:**
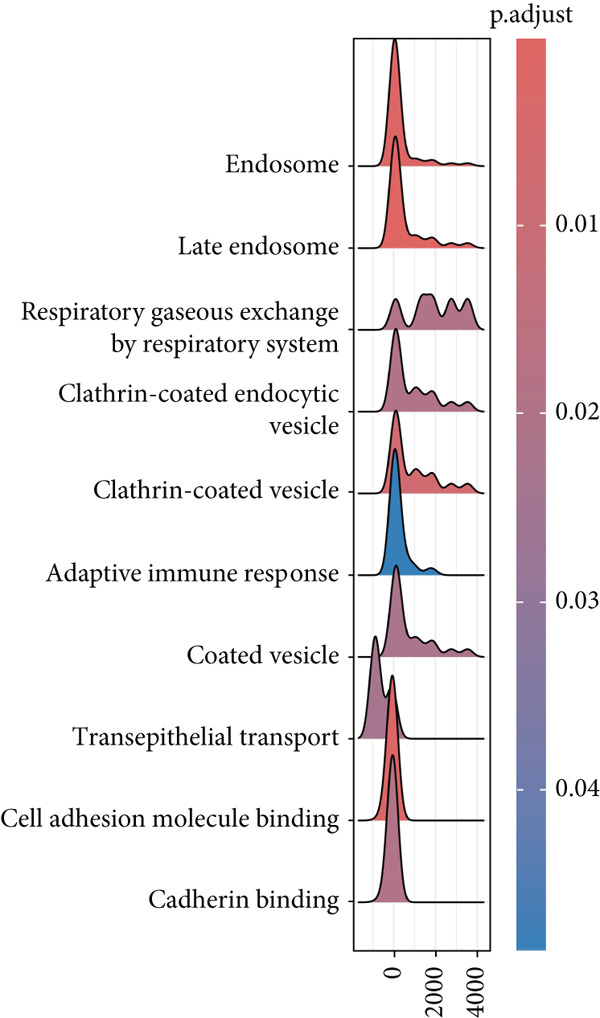
(a)

**Figure:**
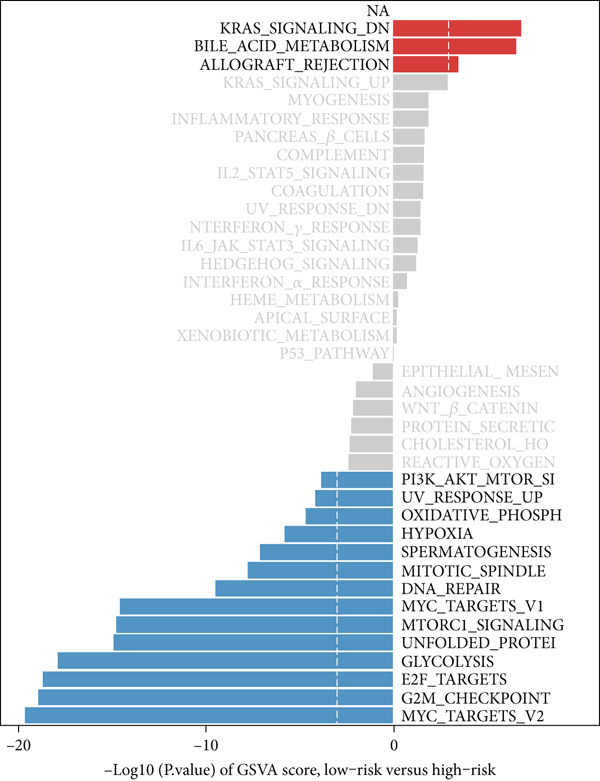
(b)

**Figure:**
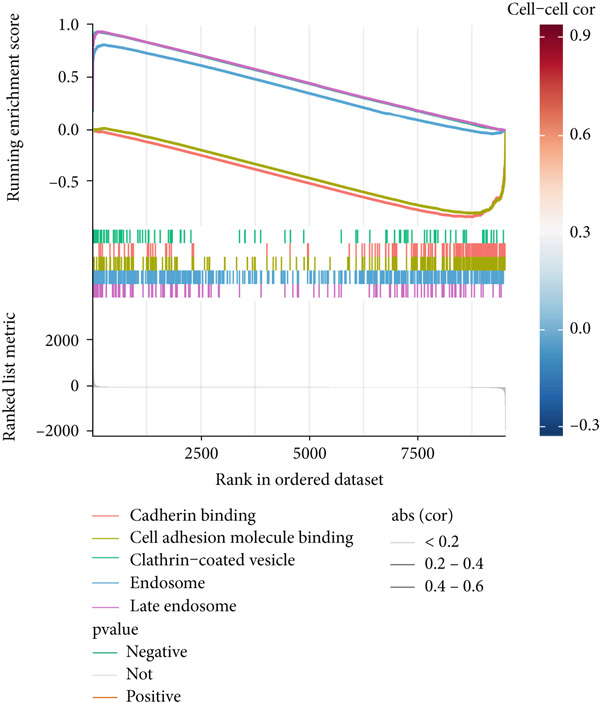
(c)

**Figure:**
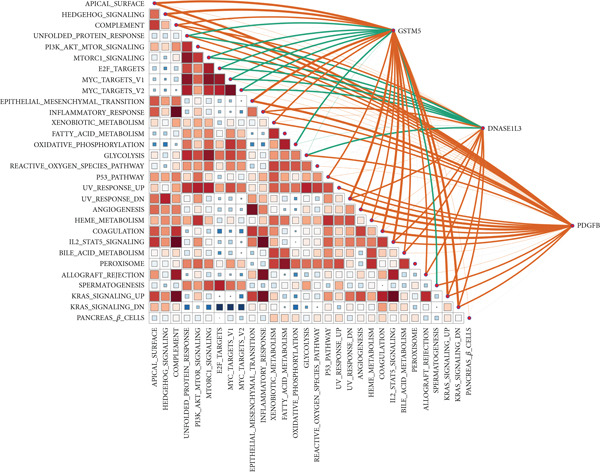
(d)

### 3.8. The Correlation Between MIGsig and Tumor–Immune Microenvironment

Utilizing the IOBR R package, a comprehensive investigation was undertaken concerning the TME in LUAD. Notably, a conspicuous association emerged wherein patients with low MIGsig levels manifested notably augmented immune cell infiltration, encompassing T cells, B cells, DCs, and macrophages, indicative of an immune activation state (Figure 8a). These discernments propose that LUADs characterized by low MIGsig levels are predisposed to classification as “hot tumors,” typified by heightened immune cell reactivity within the TME. The high‐MIGsig group displayed an immunosuppressive landscape, characterized by the accumulation of regulatory T cells, M2 macrophages, and MDSCs, along with elevated activity of EMT and other exclusion pathways (Figure 8b,c). Conversely, the low‐MIGsig group was significantly enriched for gene signatures predictive of positive immunotherapy response (Figure 8d). These results collectively indicate that while low‐MIGsig tumors possess an immunotherapy‐favorable microenvironment, high‐MIGsig tumors are dominated by an immunosuppressive and immune‐excluded phenotype.

Figure 8The TME‐related molecular characteristics of high‐ and low‐MIGsig patients. (a–d) The distribution of TME immune cell type signatures (a), immune suppression signatures (b), immune exclusion signatures (c), and immunotherapy biomarkers (d) is compared between the high‐ and low‐MIGsig patients. (e, f) The distribution of TMB and TNB is examined between the high‐ and low‐MIGsig patients. (g) The distribution of fibroblasts is analyzed between the high‐ and low‐MIGsig patients. (h) The relationship between MIGsig and fibroblasts is investigated. (i–k) Survival analysis is conducted by combining MIGsig with TMB, TNB, and fibroblasts. ns, not significant;  ^∗^ *p* < 0.05,  ^∗∗^ *p* < 0.01,  ^∗∗∗^ *p* < 0.001, and  ^∗∗∗∗^ *p* < 0.0001.

**Figure:**
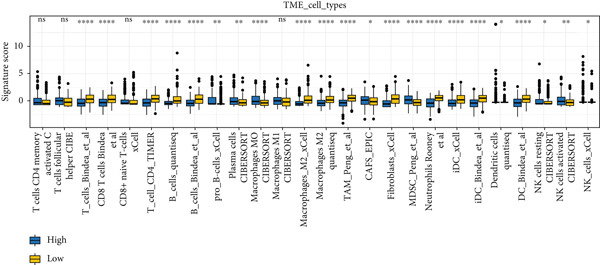
(a)

**Figure:**
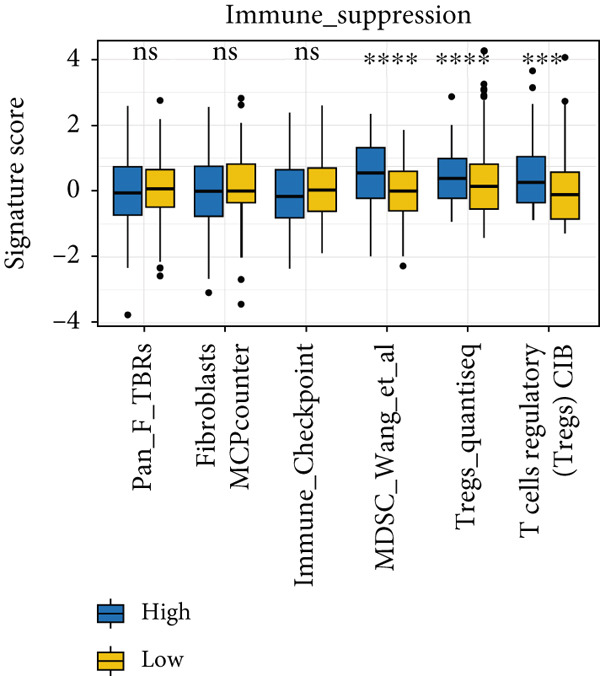
(b)

**Figure:**
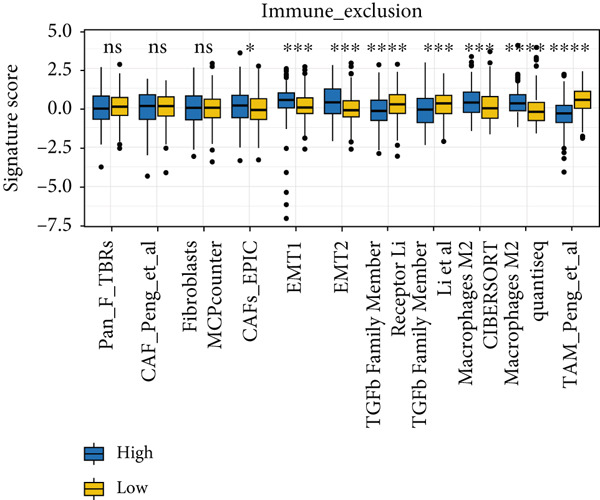
(c)

**Figure:**
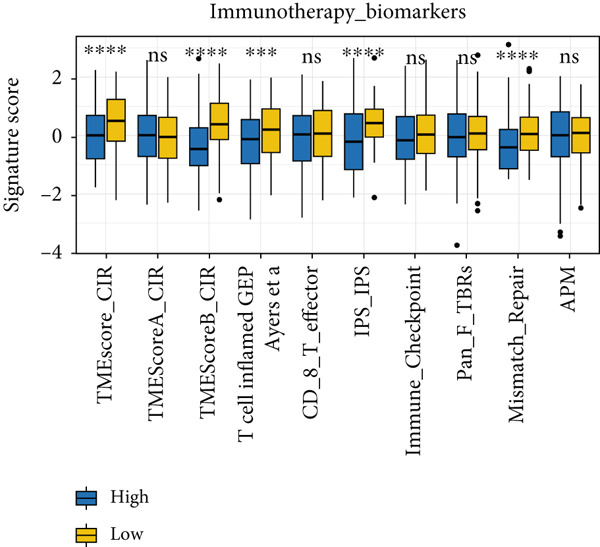
(d)

**Figure:**
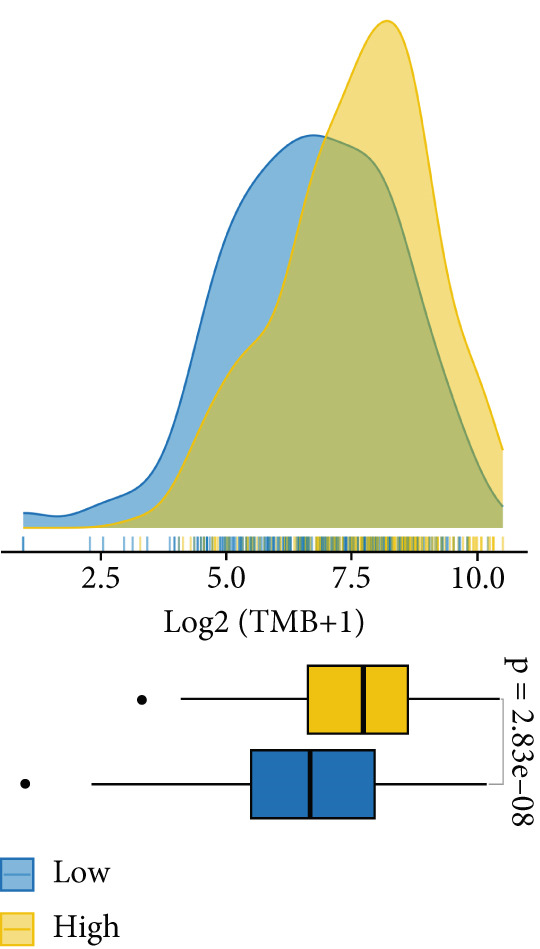
(e)

**Figure:**
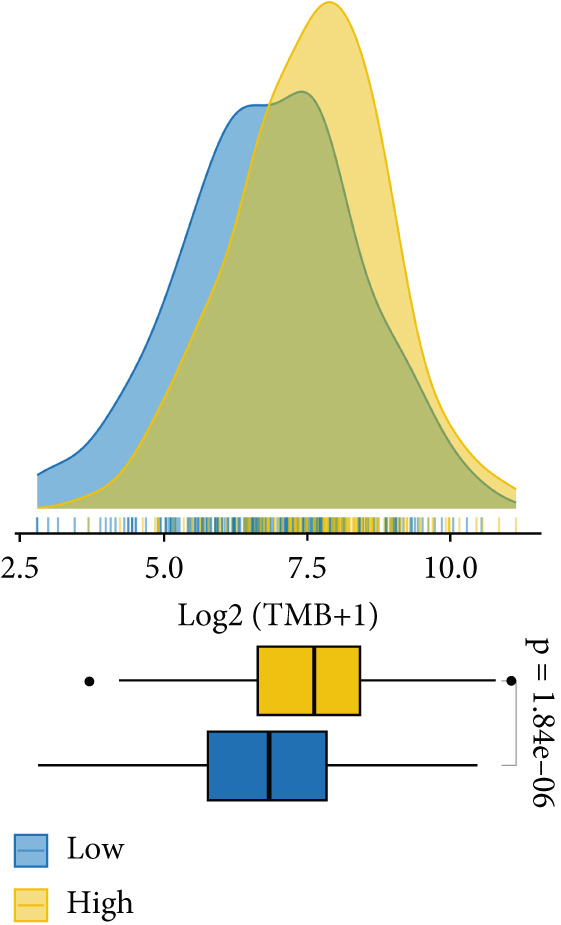
(f)

**Figure:**
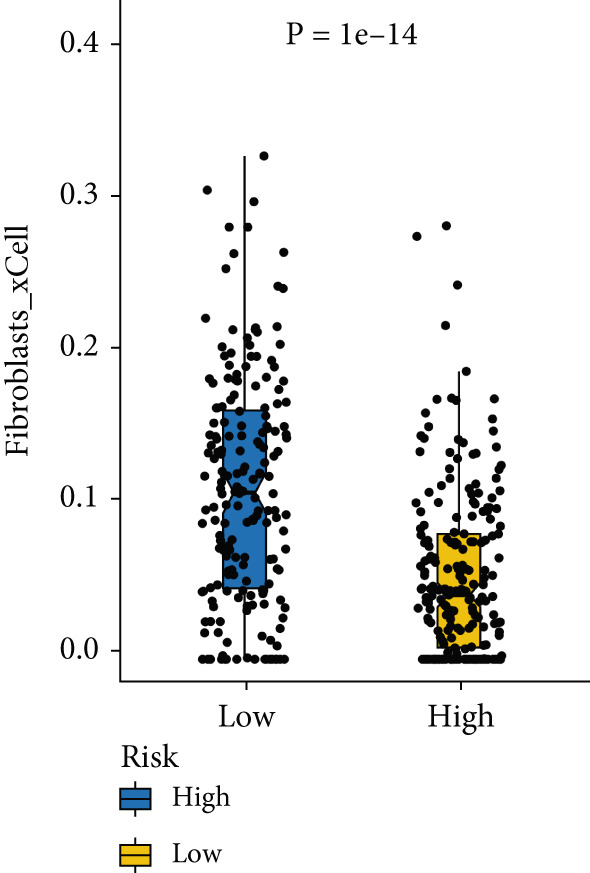
(g)

**Figure:**
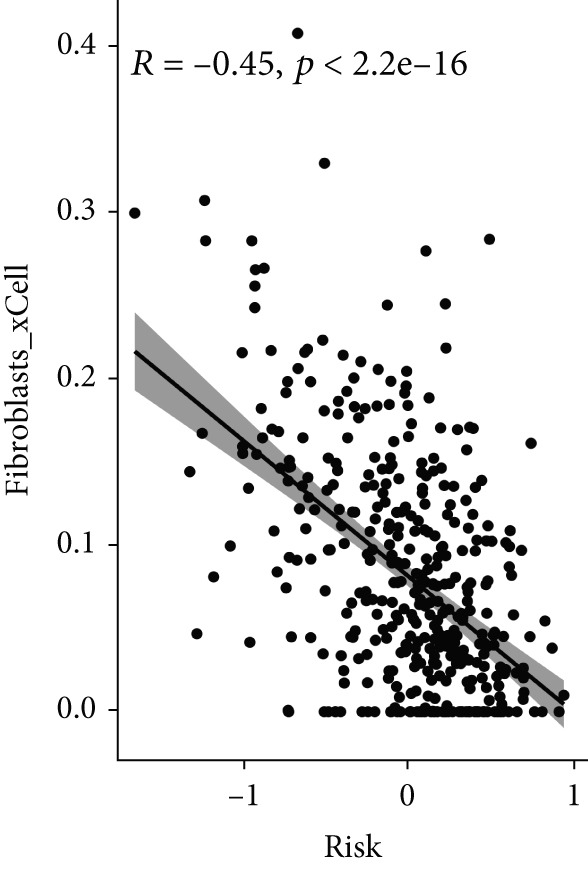
(h)

**Figure:**
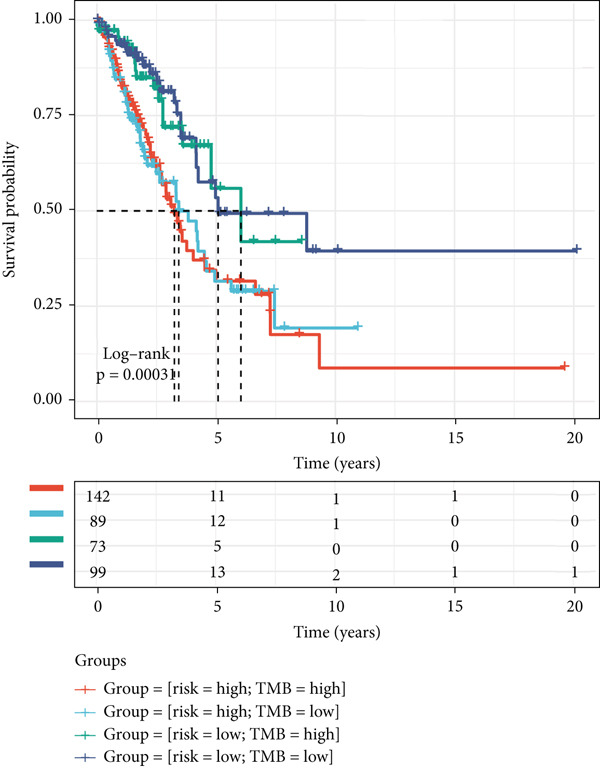
(i)

**Figure:**
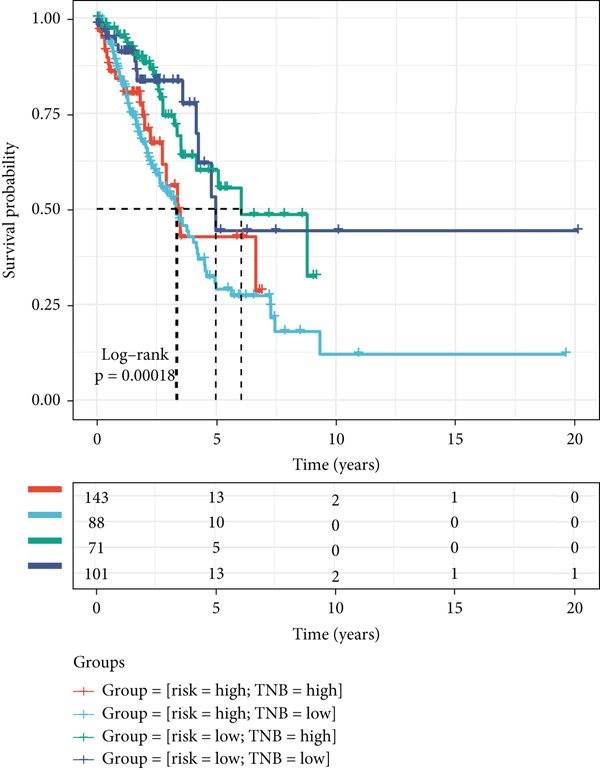
(j)

**Figure:**
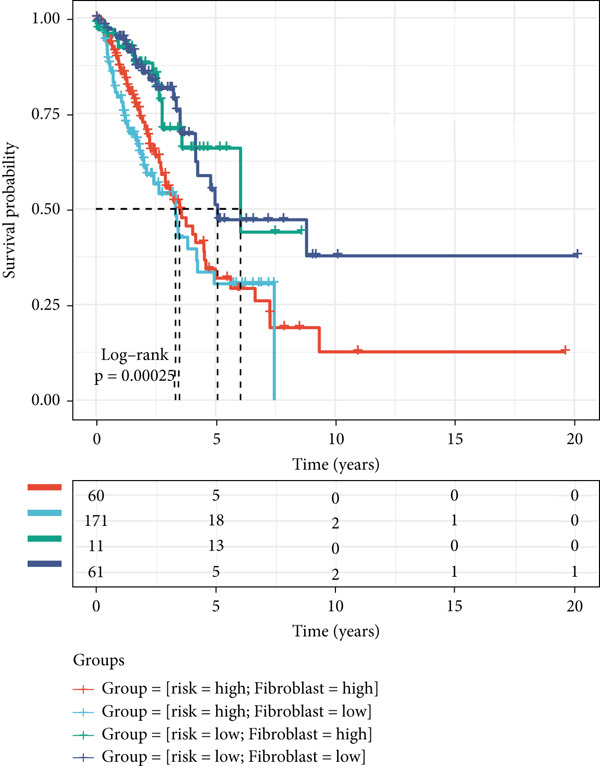
(k)

TMB and TNB serve as established biomarkers in forecasting patients′ responsiveness to immunotherapeutic interventions. Moreover, Zeng et al. shed light on the noteworthy involvement of fibroblasts in the context of immunotherapy for LUAD. Consequently, our inquiry delved into discerning variations in the expression levels of these biomarkers across distinct MIGsig cohorts. The low‐MIGsig group displayed higher enrichment of TMB, TNB, and fibroblasts, suggesting that this group may have higher immunogenicity (Figures 8e, 8f, 8g, and 8h). Survival analysis further demonstrated that the MIGsig could serve as a valuable complementary factor to TMB, TNB, and fibroblasts in differentiating patient outcomes (Figures 8i, 8j, and 8k). Notably, patients with lower MIGsig and higher TMB, TNB, or fibroblast infiltration tended to have a more favorable survival prognosis in LUAD. These results suggest that the MIGsig, along with TMB, TNB, and fibroblasts infiltration, could provide valuable prognostic information for LUAD patients.

### 3.9. The MIGsig Has Excellent Predictive Power for Immunotherapy Response

Next, we calculated the TIP score to investigate the potential biological mechanisms associated with the MIGsig. Consistent with our expectations, the low‐MIGsig group exhibited significant differences primarily in Step 2 (cancer‐associated antigen presentation), Step 4 (tumor immune‐infiltrating cell recruitment), and Step 5 (immune cell infiltration), reinforcing our previous analysis (Figure 9a). To comprehensively evaluate the contribution of MIGsig to LUAD immunotherapy, a systematic analysis was conducted, specifically targeting the IMvigor210 cohort. This cohort was chosen due to its comprehensive repository of prognostic and treatment‐related data. Unlike previous investigations, this study concentrated on discerning long‐term survival disparities among patients following a 2‐month treatment regimen (*p* < 0.05; Figure 9b). Encouragingly, the low‐MIGsig group demonstrated better prognostic outcomes, suggesting a greater therapeutic benefit from immunotherapy. These findings provide further evidence for the clinical relevance of the MIGsig in guiding immunotherapy strategies for LUAD patients.

Figure 9The value of MIGsig in predicting immunotherapy response in LUAD patients. (a) The degree of activation is compared between the high‐ and low‐MIGsig groups at each step of TIP, showcasing the differences. (b) The long‐term survival (LTS) difference is examined after 2 months of treatment between the high‐ and low‐MIGsig groups. (c) The distribution of MIGsig is analyzed among different immunotherapy response groups. (d) The subclass mapping algorithm is utilized to predict the response to immunotherapy between the high‐ and low‐MIGsig groups. (e, f) The TIDE algorithm is employed to predict the response to immunotherapy between the high‐ and low‐MIGsig groups. (g) The survival analysis is conducted to compare the high‐ and low‐MIGsig groups in the dataset GSE135222. (h) The distribution of MIGsig is examined among different immunotherapy response groups in the dataset GSE91061. ns not significant;  ^∗^ *p* < 0.05,  ^∗∗^ *p* < 0.01, and  ^∗∗∗^ *p* < 0.001.

**Figure:**
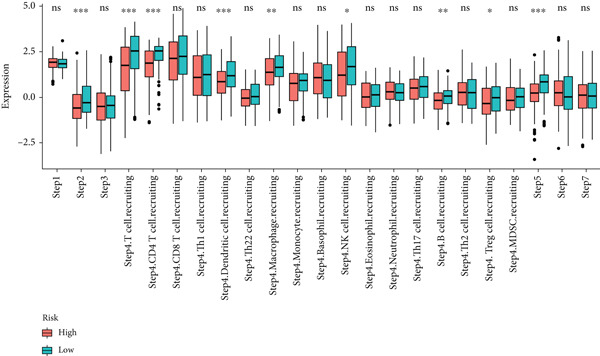
(a)

**Figure:**
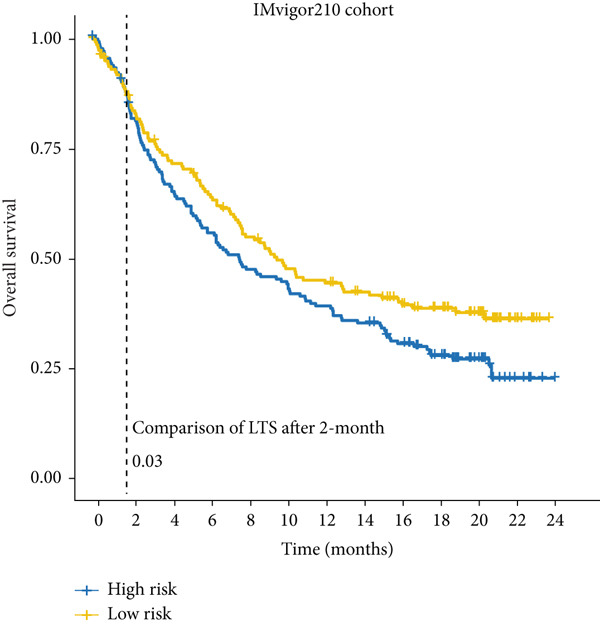
(b)

**Figure:**
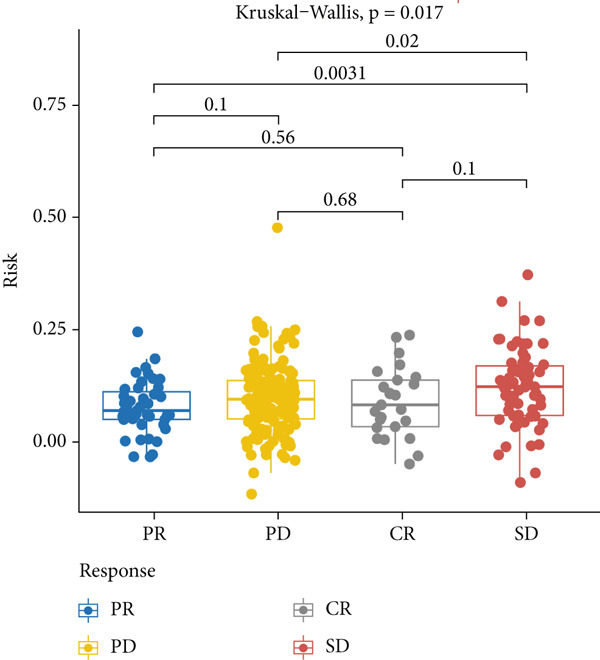
(c)

**Figure:**
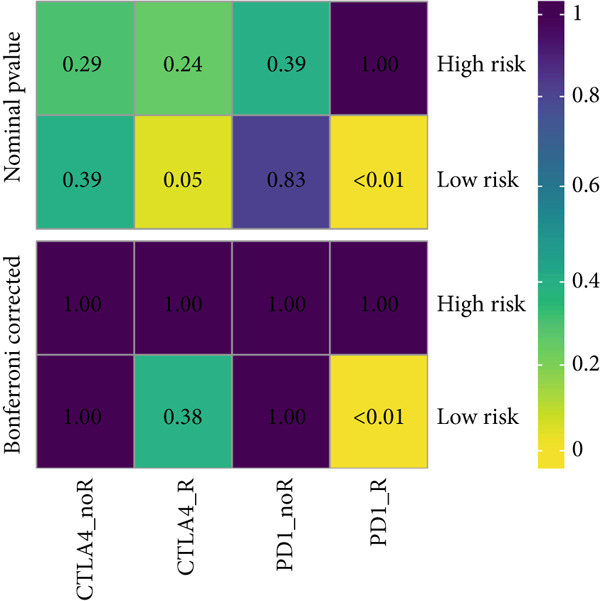
(d)

**Figure:**
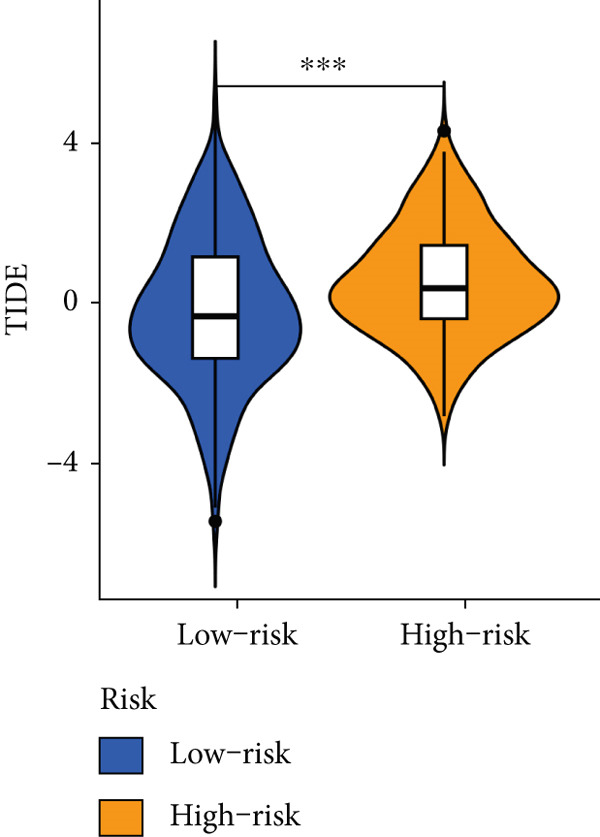
(e)

**Figure:**
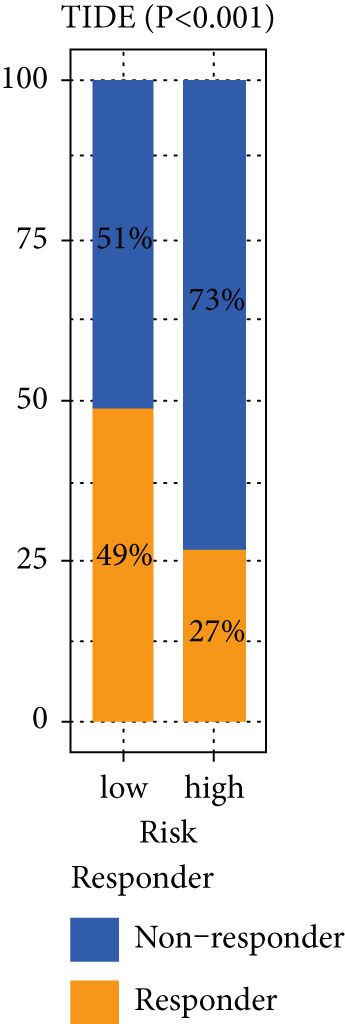
(f)

**Figure:**
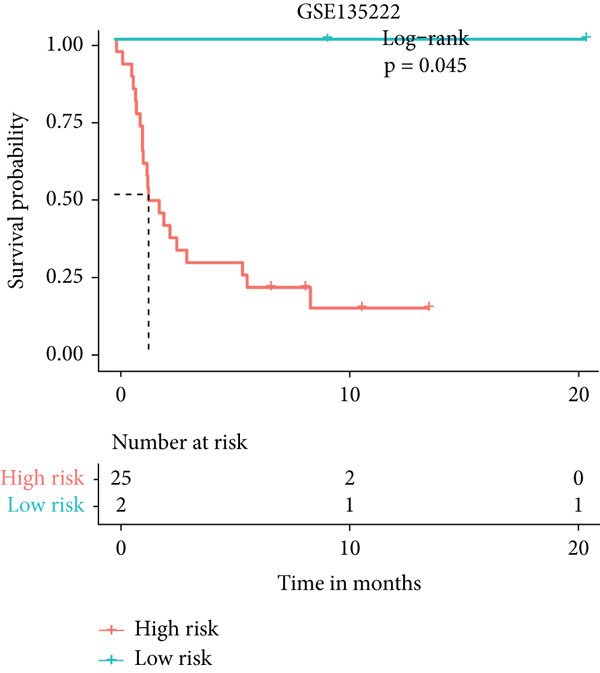
(g)

**Figure:**
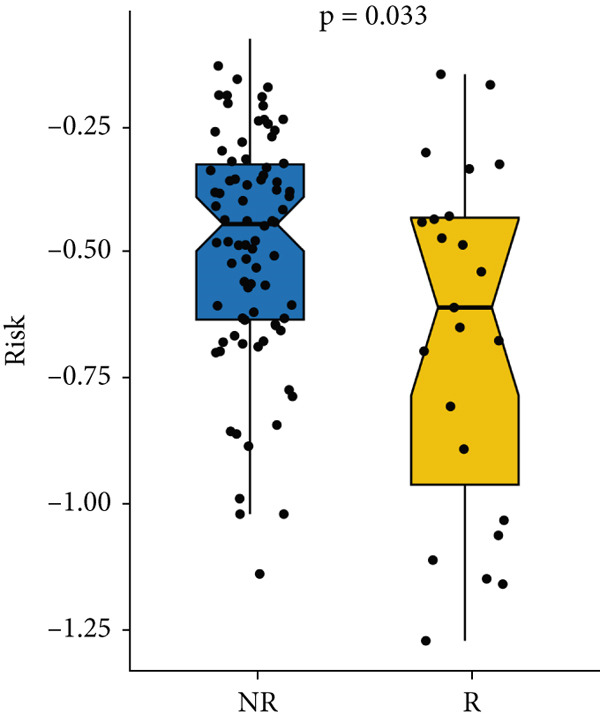
(h)

The distribution of the MIGsig among patients with different response levels further confirmed our findings. Specifically, the MIGsig score was significantly lower in the responder group (complete response [CR]/partial response [PR]) compared to the nonresponder group (progressive disease [PD]/stable disease [SD]) (*p* < 0.05; Figure 9c). Additionally, when we applied the subclass mapping algorithm to the GSE91061 cohort, we observed consistent results, where low MIGsig levels indicated a better response to PD‐1 therapy (Figure 9d). Furthermore, we utilized the TIDE algorithm to evaluate patient response to immunotherapy and observed that the low MIGsig group displayed better responsiveness (Figure 9e,f). Additionally, we conducted revalidation of our findings in two immunotherapy validation cohorts with available prognostic information. In the postimmunotherapy population, low MIGsig levels were associated with better prognostic outcomes (GSE135222, *p* = 0.045; Figure 9g). Moreover, low MIGsig levels were indicative of better immunotherapy outcomes in the GSE91061 dataset (*p* = 0.033; Figure 9h). These revalidation analyses further support the clinical significance of the MIGsig as a predictive marker for immunotherapy response and prognostic outcomes in LUAD.

### 3.10. Screening of Potential Therapeutic Drugs

Two different methodologies were employed to identify potential drug candidates that show heightened sensitivity in high‐MIGsig patients. The analyses utilized drug response data from both the CTRP and PRISM databases. Initially, we conducted a comprehensive analysis of differential drug responses, comparing the cohorts with high and low MIGsig. This analysis was aimed at identifying compounds that exhibited reduced estimated AUC values specifically within the high‐MIGsig group. Subsequently, we used the Spearman correlation coefficient to assess the association between AUC values and high‐MIGsig in order to identify compounds that exhibited negative correlation coefficients. This analysis identified a set of six compounds from the CTRP dataset: SB‐743921, paclitaxel, vincristine, KX2‐391, BI‐2536, and GSK461364 (Figure S1A). Additionally, a similar analysis using the PRISM dataset revealed another set of six compounds: PHA‐793887, docetaxel, MK‐2461, ispinesib, vincristine, and nobiletin (Figure S1B). These compounds demonstrated lower estimated AUC values in the high‐MIGsig group and were inversely correlated with the MIGsig.

### 3.11. Validation of the Prognostic Model

To validate the prognostic model comprehensively, we conducted a series of assays encompassing qRT‐PCR analysis on LUAD cell lines and tissue samples, RNA‐seq profiling on clinical tissues in the GTEx database, and evaluation of protein content via IHC. Initially, we integrated the GTEx database containing normal tissues and the TCGA database containing tumor tissues to examine mRNA expression levels (Table S3). Our analysis unveiled a notable downregulation in the expression levels of the three signature genes within tumor tissues in contrast to their expression in normal tissues (Figure 10a). A similar expression trend was observed in 20 pairs of LUAD patient samples from our hospital (Figure 10a).

Figure 10Validation of the expression patterns of three signature genes. (a) The mRNA expressions of three signature genes are compared between normal and tumor tissues. (b) The expression of the three signature genes is assessed in normal and LUAD cell lines. (c) Immunohistochemistry analysis is performed on the three signature genes in the normal and tumor groups, using data obtained from the HPA database.  ^∗∗∗^ *p* < 0.001 and  ^∗∗∗∗^ *p* < 0.0001.

**Figure:**
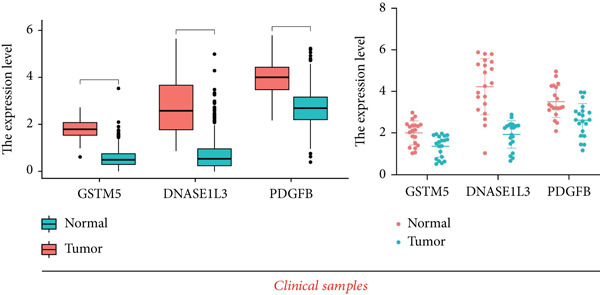
(a)

**Figure:**
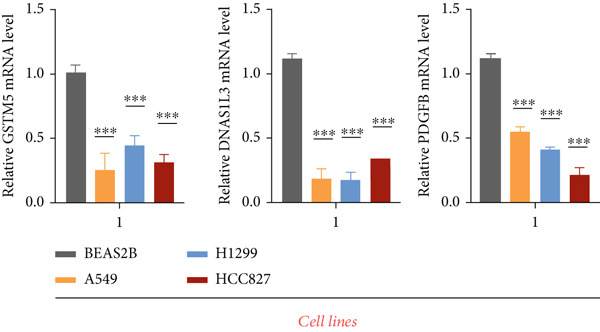
(b)

**Figure:**
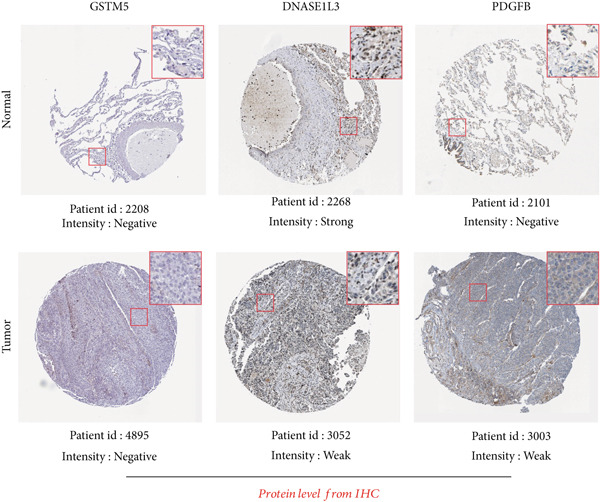
(c)

We further evaluated the expression levels of these three model genes in cell lines. This validation encompassed three distinct LUAD cell lines (A549, H1299, and HCC827) alongside a normal bronchial epithelial cell line, BEAS2B. The findings derived from these cell lines were concordant with the data gleaned from qRT‐PCR analysis of clinical tissues (Figure 10b).

Moreover, the protein expression levels of these genes were evaluated using IHC slices sourced from the HPA database. Predominantly, tumor samples demonstrated diminished protein expression relative to normal bronchial epithelial tissues, manifesting as fainter staining patterns (Figure 10c). These results further validate the accuracy of the aforementioned bioinformatics study.

## 4. Discussion

In clinical research, migrasomes have the potential to serve as biomarkers for predicting tumor progression, recurrence, and metastasis. Migrasomes released by tumor cells may contain specific tumor markers. The identification of potential tumor markers through the screening of migrasomes could offer new insights into tumor diagnosis and prognosis [16]. Additionally, migrasomes carry a variety of biomolecules. Once released into the surrounding immune microenvironment, they may act as a medium for signal exchange, participating in the shaping of the TME [17]. However, the role of migrasomes in LUAD has not been analyzed, and our research addresses this gap in knowledge.

This study employed an innovative computational framework to identify a stable and robust prognostic signature known as MIGsig. The framework integrated 10 different machine learning algorithms and explored a wide range of 101 combinations [18]. By using this sophisticated framework, we successfully reduced the complexity of the variables, optimized the model, and established a consensus model with improved predictive accuracy and translational potential. Importantly, all the genes included in the MIGsig are known to be involved in either immune response or tumor development. GSTM5 belongs to the Mu class of the glutathione S‐transferase (GST) family, situated within a 97‐kb region on chromosome 1p13 [19]. The GST gene family serves as a pivotal group of xenobiotic detoxification enzymes crucial for shielding cells against the deleterious impacts of toxic pharmaceuticals and environmental electrophiles [20, 21]. GSTM5, as a part of the GST family, has been associated with various cancer types, including breast cancer, prostate cancer, ovarian cancer, bladder cancer, and colorectal cancer [22–25]. DNASE1L3, also known as deoxyribonuclease *γ*, belongs to the Deoxyribonuclease 1 family. It is predominantly secreted by macrophages and DCs located in the liver and spleen. DNASE1L3 exhibits DNA hydrolysis activity, allowing the cleavage of both single‐ and double‐stranded DNA. This enzymatic function is essential for maintaining the homeostasis of human plasma DNA [26–28]. Previous studies have identified associations between DNASE1L3 and signaling pathways in breast cancer and the staging of renal clear cell carcinoma [29, 30]. Moreover, DNASE1L3 has been identified as a potential prognostic biomarker in LUAD and colon cancer [31, 32]. It has also demonstrated its ability to inhibit the progression of hepatocellular carcinoma by suppressing apoptosis and reprogramming glucose metabolism simultaneously [33, 34]. Platelet‐derived growth factors (PDGFs) are recognized as proangiogenic agents capable of manifesting their physiological effects via autocrine or paracrine pathways. The pivotal involvement of PDGFs in tumor angiogenesis, proliferation, invasion, and migration has been extensively substantiated in the scientific literature [35]. PDGFB is a specific subtype within the PDGF family and acts by binding to its receptor in the form of dimeric PDGF‐BB. This leads to the activation of the downstream PDGF/PDGFR signaling pathway, which can promote the malignant progression of tumors [36, 37].

The functional significance of migrasomes depends on their interaction with specific immune populations [38]. Migrasomes derived from antigen‐presenting cells have diverse actions, including the modulation, activation, or mobilization of neighboring immune cells. On the other hand, migrasomes derived from macrophages encapsulate processed antigens, enhancing their ability to recognize and interact with targets. Notably, migrasomes derived from T cells have been found to enhance cytotoxicity or suppression, depending on the specific microenvironment. These findings underscore the diverse roles of migrasomes in immune regulation, indicating their potential as promising therapeutic targets for immune‐related disorders. With this objective in mind, our study was aimed at comprehensively understanding the immune microenvironment related to the MIGsig in LUAD and elucidate its role in the antitumor immune response. Previous research has proposed the classification of solid tumors into immunoinflammatory types (commonly referred to as “hot” tumors) and immune exclusion types (commonly referred to as “cold” tumors) based on the characteristics of the TME [39, 40]. Expanding on this knowledge, our study identified significant associations between different MIGsig subgroups and the levels of immune cell infiltration within the LUAD TME. Specifically, the low‐MIGsig subgroup showed extensive enrichment of activated T cells, B cells, DCs, and macrophages, indicating an immune activation state. In contrast, the high‐MIGsig subgroup displayed high levels of infiltration by immunosuppressive cells, such as MDSCs, Tregs, and M2 macrophages. These findings suggest that the low MIGsig subtype may be associated with the characteristics of “hot” tumors, indicating potential responsiveness to immunotherapy and potentially leading to improved treatment outcomes compared to other subtypes. Significantly, we found a noteworthy enrichment of previously reported signatures associated with an improved response to immunotherapy in the low‐MIGsig group. Furthermore, through immune function and anti‐tumor immune cycle analyses, we determined that the low‐MIGsig group demonstrated enhanced cancer‐associated antigen presentation, recruitment of tumor immune‐infiltrating cells, and immune cell infiltration. Additionally, this group exhibited greater activity across most of the anticancer immune cycle steps. These findings provide further evidence of the stronger antitumor immune activity within the low‐MIGsig group, which aligns with the results obtained from functional enrichment analysis. Overall, these observations collectively support the potential of the MIGsig as a valuable biomarker for immune activity and response to immunotherapy in LUAD.

The low‐MIGsig group is characterized by higher TMB and TNB, as well as increased abundance of fibroblasts, suggesting a potential for stronger antitumor immunity. The predictive capacity of MIGsig for immunotherapeutic response was additionally corroborated through the analysis of real‐world data sourced from the IMvigor210 cohort. Importantly, patients who achieved CR/PR exhibited lower risk scores compared to those with SD/PD. These findings not only confirm the predictive capacity of the MIGsig for immunotherapy response but also suggest that the low‐MIGsig group may derive greater benefit from immunotherapy. Additionally, subclass mapping analysis demonstrated a better response to immunotherapy in the low‐MIGsig group, further supporting our findings and highlighting the potential of the MIGsig as a valuable tool for early identification of immunotherapy‐sensitive populations. Moreover, we performed an analysis using the TIDE algorithm and observed that high expression of migrasomes was associated with higher TIDE scores, indicating a greater likelihood of tumor immune escape. Migrasomes are abundant in various signaling molecules, including cytokines, growth factors, and chemokines, which are known to play crucial roles in mediating immune escape in various types of tumors [17]. Additionally, survival analysis showed that patients with low MIGsig had a more favorable prognosis. These findings have been validated across multiple immunotherapy cohorts, further supporting the significance of the MIGsig as a prognostic indicator.

Despite the promising results, there are several challenges and limitations that need to be considered. Firstly, LUAD is a highly heterogeneous disease with diverse genetic and epigenetic alterations. The heterogeneity within and between tumors may affect the generalizability of MIGsig. Therefore, further validation in larger and more diverse cohorts is necessary. Secondly, while our study highlights the importance of migrasome activity, the exact mechanisms by which migrasomes influence tumor progression and immune modulation are not fully understood. Detailed functional studies are required to elucidate these mechanisms and confirm the role of migrasomes in LUAD. Lastly, implementing MIGsig in clinical practice poses several challenges, including the standardization of gene expression assays and the interpretation of risk scores. Additionally, integrating MIGsig with existing clinical and pathological criteria will require rigorous clinical validation and the development of user‐friendly tools for clinicians.

## 5. Conclusion

In this study, we performed the first analysis of migrasomes in LUAD. Utilizing both bulk and single‐cell transcriptome data, and employing machine learning algorithms, we successfully developed and validated a migrasomes risk model. The model successfully predicted the prognosis and immunotherapy response of LUAD patients.

## Ethics Statement

This study involving human participants was conducted in accordance with the ethical principles of the Declaration of Helsinki. The study protocol was reviewed and approved by the Ethics Committee of the Second Hospital of Hebei Medical University (No. 2025‐Y173). Written informed consent was provided by the patients/participants to participate in this study.

## Conflicts of Interest

The authors declare no conflicts of interest.

## Author Contributions

J.Z.: writing (original draft), investigation, conceptualization, visualization, data curation, methodology, software, and formal analysis. T.S.: visualization, data curation, methodology, and software. C.L.: visualization, data curation, methodology, and software. N.W.: investigation and data curation. M.J.: investigation and visualization. H.G.: investigation and visualization. X.Z.: investigation and data curation. Q.F.: conceptualization, writing (review and editing), supervision, and funding acquisition. J.Z. and T.S. have contributed equally to this work and share first authorship.

## Funding

This study was supported by the Medical Science Research Project of Hebei, 20221064.

## Supporting Information

Additional supporting information can be found online in the Supporting Information section.

## Supporting information


**Supporting Information 1.** Figure S1: Potential agents for patients with high MIGsig. (a) Spearman correlation and differential response analyses of six CRTP‐derived compounds, and the difference of AUC value between high‐ and low‐risk score groups′ response to six CRTP‐derived compounds. (b) Spearman correlation and differential response analyses of six PRISM‐derived compounds, and the difference of AUC value between high‐ and low‐risk score groups′ response to six PRISM‐derived compounds.

**Supporting Information 2.** Table S1: Summary of 10 migrasome–related genes. Table S2: Primer sequences for mRNAs. Table S3: Clinical patient information.

## Data Availability

Data is available on request from the authors.
