# Supplementary figures and images for: Integrated Multiomics Analysis Reveals a Migrasome‐Related Signature for Prognosis and Immunotherapy Response in Lung Adenocarcinoma
Source: Hum Mutat. 2026 Jan 8;2026:8778797. doi: 10.1155/humu/8778797 (PMC12781864; doi:10.1155/humu/8778797)

A

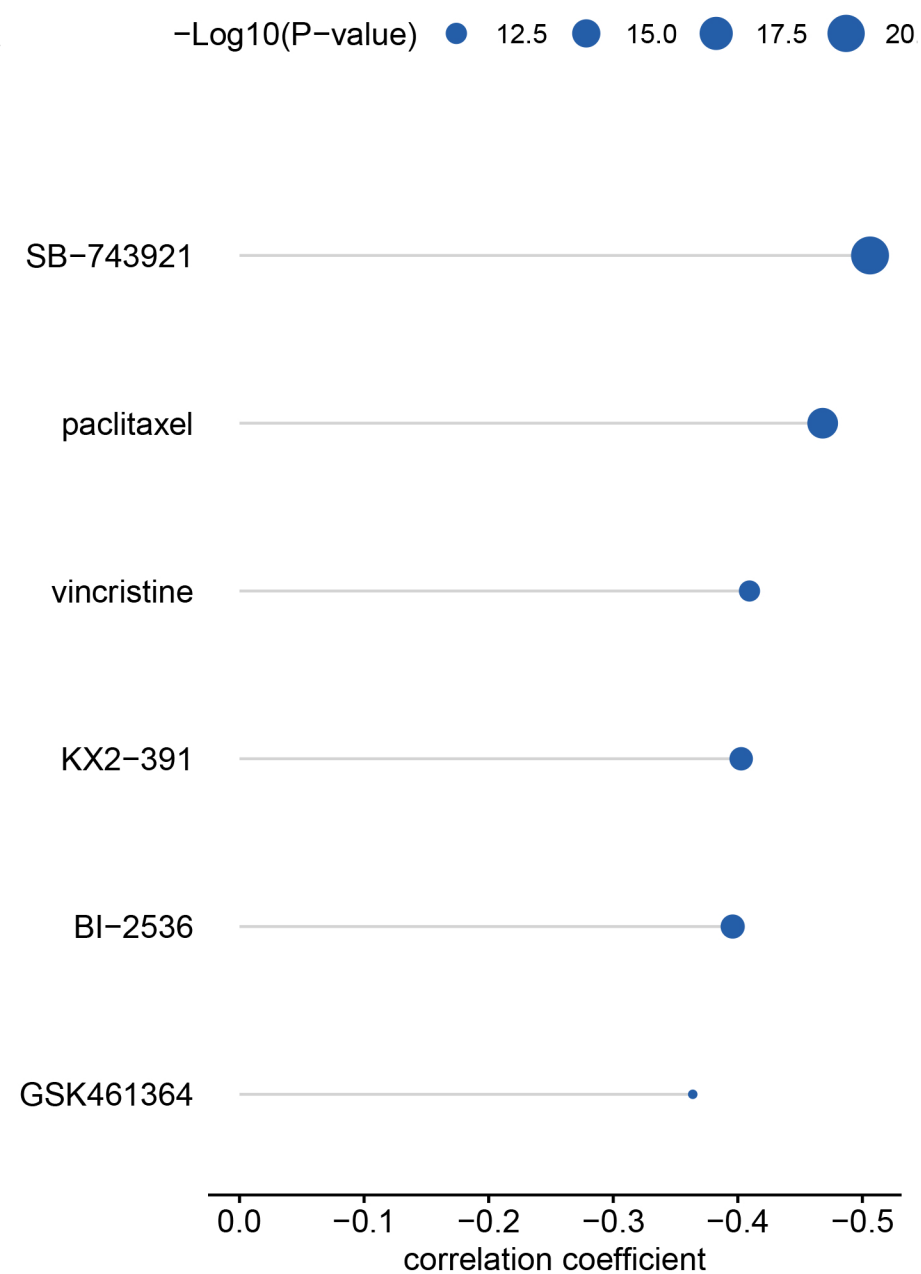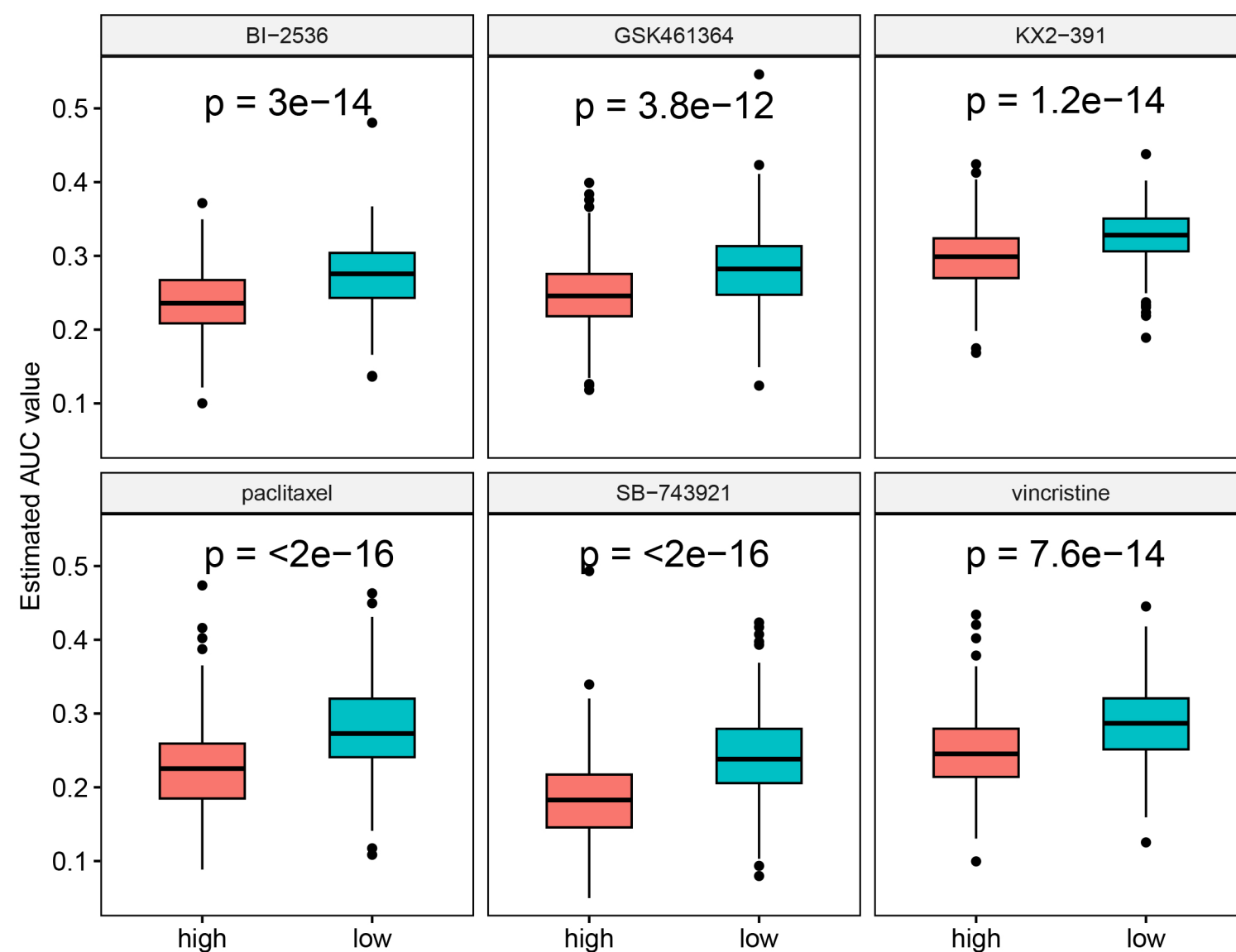

B

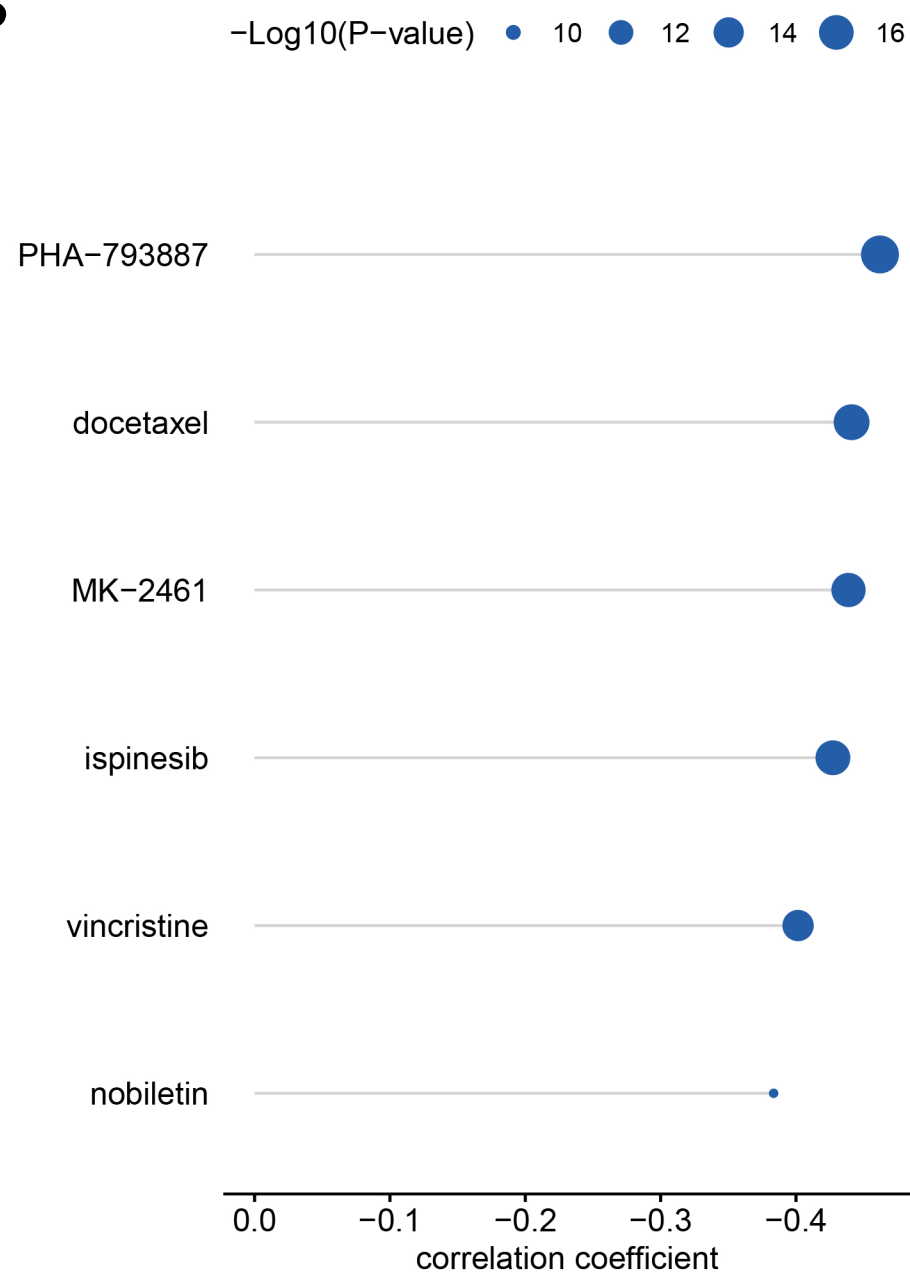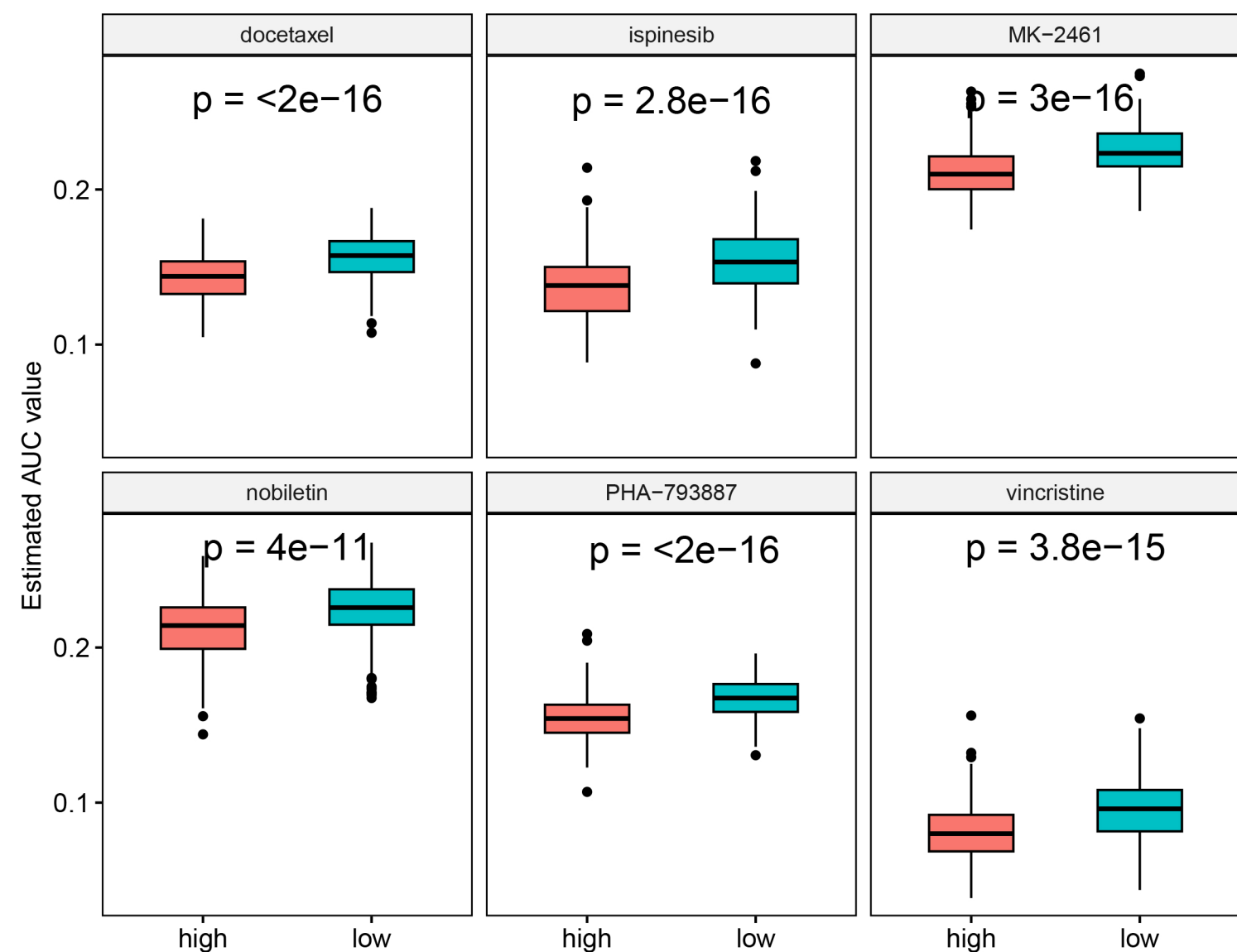

Supplement: Supplementary file 1 — Supporting Information 1. Figure S1: Potential agents for patients with high MIGsig. (a) Spearman correlation and differential response analyses of six CRTP‐derived compounds, and the difference of AUC value between high‐ and low‐risk score groups′ response to six CRTP‐derived compounds. (b) Spearman correlation and differential response analyses of six PRISM‐derived compounds, and the difference of AUC value between high‐ and low‐risk score groups′ response to six PRISM‐derived compounds. [file HUMU-2026-8778797-s002.pdf]
